# Propolis-Based Microneedles Incorporating Neratinib-Loaded *Ocimum basilicum* Essential Oil-Based Lipid Nanovesicles for Minimally Invasive Cancer Therapy: Formulation and In Vivo Evaluation in an Ehrlich Solid Tumor Model

**DOI:** 10.3390/pharmaceutics18101244

**Published:** 2026-09-30

**Authors:** Sammar Fathy Elhabal, Suzan Awad AbdelGhany Morsy, Marian Sobhy Azer, Khaled M. Allam, Fatma E. Hassan, Tassneim M. Ewedah, Rania Mostafa Abd El Galil, Mahmoud Nour El-Din, Ahmed I. Ashmawy, Heba Sabry Ahmed, Azza ElSayed Abd ElFattah Mansy, Ahmed Mohsen Elsaid Hamdan

**Affiliations:** 1 Department of Pharmaceutics and Industrial Pharmacy, Faculty of Pharmacy, Modern University for Technology and Information (MTI), Mokattam, Cairo 11571, Egypt; 2 Department of Clinical Pharmacology, Faculty of Medicine, Alexandria University, Alexandria 21526, Egypt; susan.ulghani@alexmed.edu.eg; 3 Pathological Sciences Department, MBBS Program, Fakeeh College for Medical Sciences, Jeddah 21461, Saudi Arabia; 4 Department of Pharmaceutics, College of Pharmaceutical Sciences and Drug Manufacturing, Misr University for Science and Technology (MUST), 6th of October City 12566, Egypt; marian.sobhy@must.edu.eg (M.S.A.); rania.mohamed@must.edu.eg (R.M.A.E.G.); 5 Department of Pharmacognosy, Faculty of Pharmacy, Qena University, Qena 83523, Egypt; khaled.mohamed@svu.edu.eg; 6 Department of Physiology, General Medicine Practice Program, Batterjee Medical College, Jeddah 21442, Saudi Arabia; fatma.e.elsayed@kasralainy.edu.eg; 7 Medical Physiology Department, Faculty of Medicine, Cairo University, Kasr Alainy, Giza 11562, Egypt; 8 Pharmaceutics and Pharmaceutical Technology Department, Faculty of Pharmacy, Egyptian Russian University, Cairo 11829, Egypt; tassneim-mohammed@eru.edu.eg; 9 Department of Pharmacology and Toxicology, Faculty of Pharmacy, University of Sadat City, Menoufia 32897, Egypt; mahmoud.noureldin@fop.usc.edu.eg; 10 Department of Pharmacology and Therapeutics, Faculty of Pharmacy, Pharos University in Alexandria (PUA), Alexandria 21311, Egypt; ahmed.ashmawy@pua.edu.eg; 11 Department of Clinical Pharmacology, Faculty of Medicine, Zagazig University, Zagazig 44519, Egypt; hsahmad@medicine.zu.edu.eg; 12 Department of Clinical Pharmacy, Faculty of Pharmacy, Menoufia University, Menoufia 32811, Egypt; azza.mansy@phrm.menofia.edu.eg; 13 Department of Pharmacology and Toxicology, Faculty of Pharmacy, University of Tabuk, Tabuk 71491, Saudi Arabia; 14 Prince Fahad bin Sultan Chair for Biomedical Research (PFSCBR), Tabuk 74191, Saudi Arabia

**Keywords:** neratinib, *Ocimum basilicum* oil, nanovesicles, propolis, dissolving microneedles, breast cancer, cytotoxicity, Ehrlich solid carcinoma

## Abstract

**Background**: Neratinib (NTB) is a potent pan-HER inhibitor, but its oral bioavailability is limited due to poor aqueous solubility, low permeability, and gastrointestinal toxicity. This study incorporated a bioactive transdermal system by incorporating NTB-loaded nanovesicles of *Ocimum basilicum* oil (NTB-OOs) into dissolving microneedles of PVA/PVP–propolis. **Methods**: *Ocimum basilicum* oil was characterized by gas chromatography–mass spectrometry (GC–MS). NTB-OOs were screened by I-optimal design and evaluated for vesicular properties, stability, release, skin permeation, and cytotoxicity. The optimized vesicles were loaded in propolis microneedles and evaluated mechanically, ex vivo, and in MCF-7 cells and Ehrlich solid carcinoma-bearing mice. **Results:** GC–MS revealed a volatile profile rich in linalool, methyl jasmonate, and a cyclopenta[g]-2-benzopyran derivative with reported antioxidant and anticancer activities. The optimized NTB-OOs showed a particle size of (198.9 ± 0.98 nm), entrapment efficiency of (88.9 ± 0.45%), PDI of (0.16 ± 0.01), zeta potential of (−23.1 ± 0.35 mV), and six-month stability. The microneedles provided zero-order release (48–50% after 24 h) and ex vivo permeation (~3500 µg/cm^2^), which were 2.7- and 1.2-fold higher than those of the NTB suspension and NTB-OOs, respectively. NTB-OOs reduced MCF-7 IC_50_ from 11.1 to 5.1 µg/mL. In vivo, NTB-OOs/propolis microneedles achieved 75.6% tumor inhibition, increased mean survival from 19 to 26.5 days, and raised the T/C ratio to 139.5%. They also induced apoptosis and downregulated the expression of HER-2, EGFR, VEGF-A, and Bcl-2. **Conclusions:** The multifunctional nanovesicle–propolis microneedle platform improves NTB solubilization, controlled release, dermal delivery, and antitumor efficacy, supporting its potential for minimally invasive localized breast cancer therapy.

## 1. Introduction

Breast cancer (BC) is the most frequently diagnosed malignancy in women and is strongly influenced by hormonal, genetic, lifestyle, and environmental factors [1,2,3]. Its biological heterogeneity necessitates innovative anticancer strategies, which can be tested in experimental models that are indispensable [4]. MCF-7 is a commonly used cell line model of estrogen receptor (ER)-positive breast cancer. Ehrlich ascites carcinoma (ESC) is a murine mammary adenocarcinoma that occurs spontaneously and is commonly used in vivo because of its rapid growth, inflammatory environment, and ability to enhance vascular permeability and tumor progression [5,6,7]. Traditional breast cancer treatments, such as surgery, radiation, chemotherapy, hormonal therapy, and targeted therapy, have advanced [8]. Their effectiveness is often undermined by systemic toxicity, drug resistance, and inadequate patient adherence. Thus, current research focuses on developing advanced pharmaceutical delivery systems and natural bioactive compounds to enhance the efficacy of anticancer drugs and reduce adverse effects caused by their use [9,10].

Neratinib (NTB) has also been investigated in HER2-mutant solid tumors [11]. It is a tyrosine kinase inhibitor used for breast cancer therapy, but these studies are mainly in clinical trials or for investigational use, not the main approved indication [12]. Studied tumor types include biliary tract cancer/cholangiocarcinoma, gallbladder cancer, cervical cancer, lung cancer, colorectal cancer, bladder cancer, and salivary gland cancer [13,14]. Activity appears stronger in some HER2-mutant biliary cancers and weaker or variable in others. NTB is an irreversible pan-HER tyrosine kinase inhibitor primarily used for HER2-positive breast cancer, including extended adjuvant treatment of early-stage disease and, in combination with capecitabine, treatment of previously treated advanced or metastatic HER2-positive breast cancer [15,16]. Despite its therapeutic potential, NTB is classified as a BCS class IV drug, with poor aqueous solubility, low permeability, slow dissolution, and variable oral bioavailability [17]. The oral therapy currently on the market requires a relatively high daily dose, which may be associated with decreased patient compliance and gastrointestinal adverse effects [18,19]. Thus, the development of alternative delivery platforms may be beneficial for augmenting the therapeutic efficacy of NTB while minimizing systemic side effects [20,21]. These platforms should be capable of enhancing NTB solubilization, permeation, and localized delivery.

*Ocimum (basil)* is a genus of aromatic medicinal plants used in folk medicine, medicine, cosmetics, and cooking. Phytochemical profiles and essential oils of important species such as *Ocimum basilicum*, *O. tenuiflorum*, and *O. gratissimum* are highly [22,23,24]. The profile includes lipophilic volatiles, oxygenated monoterpenes, terpenoid esters, phenolic compounds, and esterified bioactives. *Ocimum* oil has been reported to contain many important compounds, including linalool, methyl eugenol, linalyl butyrate, methyl jasmonate, 2-methyl-2-octanol, and lactone or methyl ester derivatives [25,26]. These components enhance the antioxidant, antimicrobial, anti-inflammatory, aromatic, and skin-conditioning properties of *Ocimum* oil, which are important in pharmaceuticals and cosmeceuticals. However, its effectiveness in topical applications may be limited, and its incorporation into hydrophilic systems may be hindered by high volatility, lipophilicity, poor water solubility, and potential instability. The use of *Ocimum* oil in nanovesicular systems, along with an edge activator and cholesterol, appears to be a promising strategy to enhance local delivery, membrane integrity, vesicle flexibility, and skin penetration [27,28].

Microneedles have emerged as a promising minimally invasive approach for transdermal and intradermal drug delivery [29,30]. MNs can penetrate the stratum corneum and create transient microchannels within the epidermis [31,32]. This approach enables the delivery of therapeutic substances with less patient discomfort and improved compliance [33]. The main water-soluble and biocompatible polymers used to make dissolving microneedles are polyvinyl alcohol, polyvinyl pyrrolidone, hyaluronic acid, carboxymethyl cellulose, and gelatin. These polymers exhibit rapid dissolution, high drug-loading capacity, and good mechanical integrity on the skin surface [34,35]. In addition to polymers, bioactive functional components can be incorporated into microneedle matrices to enhance therapeutic efficacy [36,37]. Propolis, a naturally occurring bioactive compound, may be beneficial [38,39]. It is a viscous material comprising a complex mixture of bioactive phytochemicals, including flavonoids, phenolic acids, phenolic esters, and terpenoids [40,41]. Propolis is often used in microneedle formulations and may confer antioxidant, antimicrobial, anti-inflammatory, and wound-healing effects that may be additive [42,43].

This study aimed to develop, optimize, and evaluate a novel propolis-assisted dissolving microneedle (MN) platform loaded with nanovesicles of *Ocimum* oil containing neratinib (NTB) for transdermal anticancer delivery. To the best of our knowledge, this is the first study to combine *Ocimum* oil-based elastic nanovesicles with propolis-loaded polymeric MNs for NTB delivery in breast cancer treatment. The proposed system aims to leverage the multifunctional role of propolis in the microneedle matrix, the vesicular stabilization and drug-entrapment potential of cholesterol/edge-activator-based nanovesicles, and the permeation-enhancing and bioactive properties of *Ocimum* oil. The vesicle properties, mechanical performance of the microneedles, drug content, dissolution kinetics, in vitro release, ex vivo skin permeation, cytotoxicity against MCF-7 cells, and in vivo antitumor efficacy of the optimized formulation were systematically characterized in the Ehrlich solid carcinoma model. The integrated nanovesicular microneedle approach is expected to enhance the transdermal delivery of NTB, improve localized tumor targeting, overcome the limitations of conventional oral therapy, and provide a promising, minimally invasive platform for breast cancer treatment.

## 2. Materials and Methods

### 2.1. Chemicals and Reagents

Fresh aerial parts (leaves and flowering tops) of *Ocimum basilicum* L. were collected from the Medicinal Plants Garden at the Faculty of Pharmacy, Qena University, Egypt, in June 2023. Neratinib (NTB, ≥98% pure), D-α-tocopheryl polyethylene glycol 1000 succinate (TPGS), Tween 80, sodium cholate, cholesterol, polyvinyl alcohol (PVA), polyvinylpyrrolidone K90 (PVP K90), propolis, hydroxypropyl methylcellulose (HPMC), mannitol, and polydimethylsiloxane (PDMS) microneedle molds were obtained from Sigma-Aldrich (St. Louis, MO, USA). Orthophosphoric acid, potassium bromide, phosphotungstic acid, phosphate-buffered saline components, and other solvents and chemicals were purchased from VWR in Milan, Italy. Parafilm^®^ M was obtained from BRAND GMBH (Wertheim, Germany). Dulbecco’s Modified Eagle’s Medium, fetal bovine serum, penicillin–streptomycin, MTT reagent, and doxorubicin were obtained from Mumbai, Maharashtra, India. Distilled water and acetonitrile (HPLC grade) were obtained from Merck Life Sciences. The human breast adenocarcinoma MCF-7 cell line was purchased from the American Type Culture Collection (ATCC, Manassas, VA, USA). Commercial ELISA kits for caspase-3, vascular endothelial growth factor, tumor necrosis factor-α, interleukin-6, malondialdehyde, catalase, and troponin-1 were obtained from LifeSpan Biosciences, Inc. (Seattle, WA, USA). QIAzol reagent was purchased from Qiagen (Hilden, Germany); the SensiFAST™ cDNA Synthesis Kit from Bioline (London, UK); and HERA SYBR Green PCR Master Mix from Willowfort (Nottingham, UK).

### 2.2. Authentication and Preparation of Plant Material

The plant material was authenticated by a taxonomist in the Department of Pharmacognosy, Faculty of Pharmacy, Qena University. A voucher specimen (No. Qn-Ph-Cog-027) was deposited in the departmental herbarium for future reference. The collected plant material was thoroughly washed with distilled water to remove dust and foreign matter, then air-dried in the shade at room temperature (25 ± 2 °C) in a well-ventilated area for 7–10 days until a constant weight was achieved. The dried material was ground into a coarse powder using a laboratory grinder and stored in airtight, amber-colored glass containers at room temperature until extraction.

### 2.3. Extraction of Essential Oil of Ocimum basilicum L.

The essential oil was extracted from the dried aerial parts by hydrodistillation using a Clevenger-type apparatus (Quickfit^®^, Borosilicate Glass, UK). Briefly, 200 g of air-dried, powdered plant material was transferred to a 5 L round-bottom flask, and 2.0 L of distilled water was added (plant material:water ratio = 1:10, *w*/*v*). The mixture was allowed to soak for 30 min before heating to ensure complete hydration of the plant tissues. Hydrodistillation was performed using an electrically controlled heating mantle. The heating rate was gradually adjusted until gentle boiling was achieved in approximately 20–30 min. Thereafter, distillation was maintained at a constant boiling rate throughout the extraction period [44,45,46]. Distillation continued for 3 h, calculated from the appearance of the first condensate drop in the graduated receiver of the Clevenger apparatus. The condensed vapors were continuously separated into aqueous and oily phases. The essential oil accumulated in the graduated arm of the Clevenger apparatus and was carefully collected using a glass Pasteur pipette. Residual moisture was removed by drying the oil over anhydrous sodium sulfate (Na_2_SO_4_) for 24 h at 4 °C. The dried oil was filtered through Whatman No. 1 filter paper and transferred into sterile amber glass vials with Teflon-lined screw caps. The samples were stored at 4 °C in darkness until GC–MS analysis.

### 2.4. GC/MS Analysis of Ocimum basilicum Oil

The chemical composition of the isolated *Ocimum basilicum* oil was evaluated using a Trace GC–TSQ mass spectrometer system (Thermo Scientific, Austin, TX, USA) fitted with a TG-5MS fused-silica capillary column (30 m × 0.25 mm i.d., 0.25 µm film thickness). The oven temperature was initially set at 50 °C and held for 4 min, then increased to 250 °C at a programmed rate, and finally to 300 °C and held for 2 min. The injector and MS transfer-line temperatures were maintained at 270 °C and 260 °C, respectively. The carrier gas was helium at a steady flow rate of 1 mL/min. Then, 1 µL of a diluted sample of the separated oil was automatically injected into the GC system in split mode using an AS1300 autosampler. A 4 min solvent delay was used. Mass spectra were obtained in full-scan mode using electron ionization (EI) at 70 eV, spanning an m/z range of 50–650. The ion-source temperature was maintained at 200 °C. The volatile and semi-volatile ingredients of *O. basilicum* oil were identified by comparing their mass spectra with those in the NIST 14 and WILEY 09 mass spectral libraries. The relative fraction of each detected compound was computed using GC peak area normalization.

### 2.5. Analytical Method for Neratinib Quantification by HPLC–UV

In vitro quantification of NTB in the samples was performed using HPLC–UV (high-performance liquid chromatography with ultraviolet detection), a validated method. The method was used to determine the entrapment efficiency (% EE), the concentration of NTB in in vitro drug-release samples, and the drug content in stability-study samples. Chromatographic separation was performed on a Phenomenex Kinetex C18 column (250 mm × 4.6 mm i.d., 5 μm). The analysis was performed by isocratic elution with a mobile phase consisting of methanol in water at a 40:60 (*v*/*v*) ratio, containing 0.1% (*v*/*v*) orthophosphoric acid (pH 2.5 ± 0.1) [47,48,49]. The mobile phase was delivered at a flow rate of 1.0 mL/min. The detection wavelength was 268 nm, and the column temperature was maintained at 25 °C. Then, 10 µL of the prepared specimen was injected into the HPLC apparatus for each analysis. The precision, accuracy, and linearity of the method were partially validated. The calibration curve exhibited linearity within the concentration range of 1000 to 15,000 ng/mL. The limit of quantification was 46 ng/mL. The validated HPLC–UV method was successfully applied to accurately determine NTB in formulation analysis, release studies, and evaluation.

### 2.6. Animals

Forty female albino mice (10–12 weeks old, 25–35 g) were obtained from the Animal Facility at the Medical Research Institute, Alexandria University. Mice were housed in plastic cages with free access to food and water at 20  ±  2 °C, 55  ±  5% relative humidity, and a 12 h light/dark cycle. The Experimental Protocol was approved by the Research Ethics Committee of Cairo University’s Faculty of Pharmacy (PI 4097). At the end of the treatment period, mice were fasted overnight and locally anesthetized with a mixture of Alcaine and BNP ophthalmic anesthetics. Heparinized capillary tubes were used to collect 0.5 mL of blood from the retro-orbital plexus, and samples were transferred into EDTA tubes placed on ice. The mice were then anesthetized with xylazine (1 mg/kg) and ketamine (75–100 mg/kg) before decapitation. Breast tumors were removed, numbered, weighed, and measured with a precision caliper to 1 mm resolution. They were then prepared for histological studies.

### 2.7. Methods

#### 2.7.1. Design of Experiments: Neratinib-Loaded Ocimum Oil Nanovesicle Formulations (NTB-OOs)

Neratinib-loaded *Ocimum* oil nanovesicle formulations (NTB-OOs) were prepared using a Custom I-optimal experimental design with 3 independent variables at 3 levels to assess how the factors affect vesicle properties. This design reduces the number of experimental runs, lowers costs, and saves time. Table 1 lists the factors studied, their levels, and constraints. The three independent variables were the amounts of Edge activator (mg) (A), Cholesterol (mg) (B), and type of edge activator (C). The responses examined were entrapment efficiency (%) (EE%), vesicle size (PS), polydispersity index (PDI), and zeta potential (ZP). The optimized NTB-OOs formula was selected for its highest desirability using Design-Expert^®^ software (Version 13, Stat-Ease, Inc., Minneapolis, MN, USA) as shown in Table 2.

#### 2.7.2. Preparation and Optimization of Neratinib-Loaded Ocimum Oil Nanovesicle Formulations (NTB-OOs)

**Figure 1:**
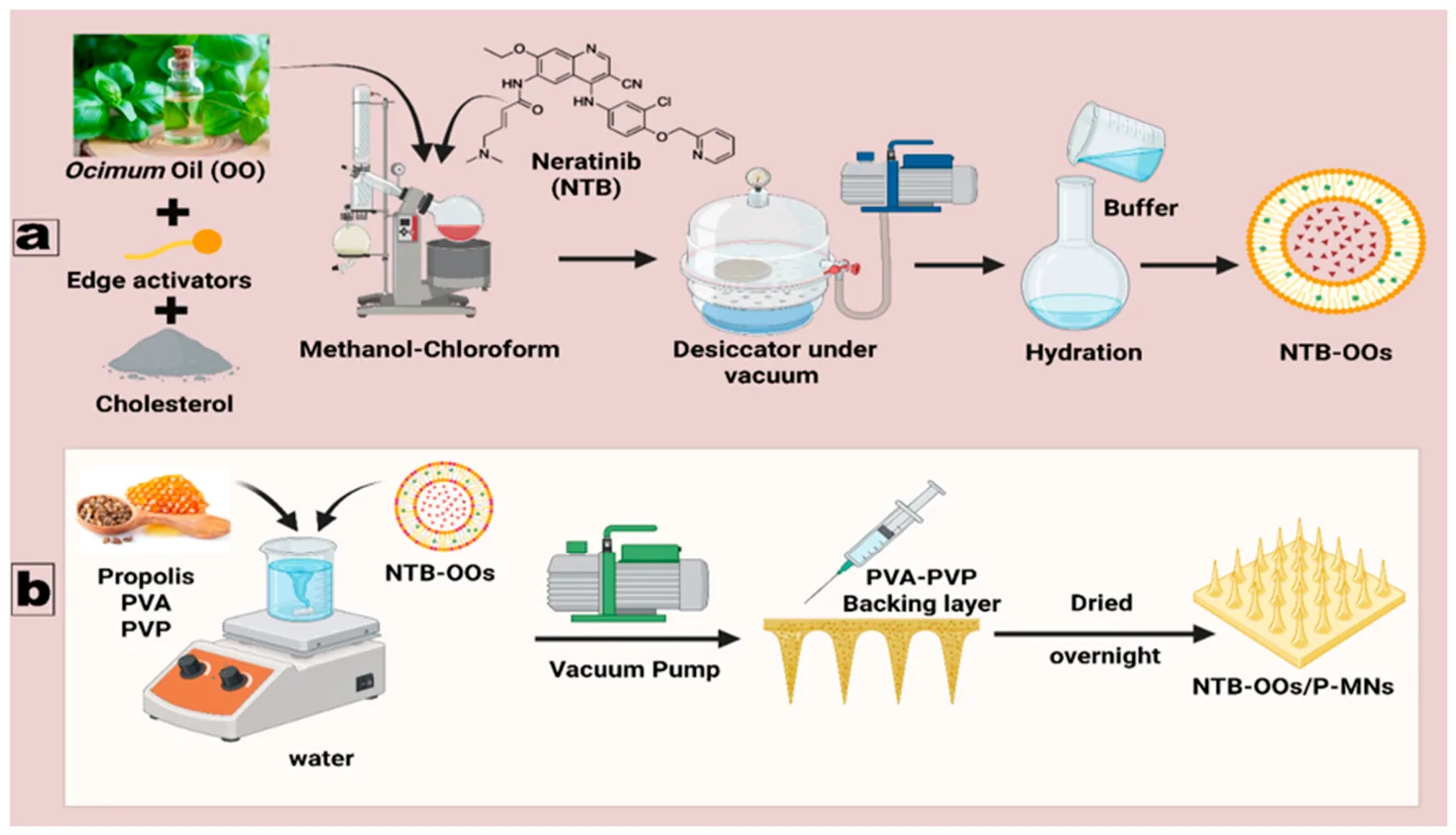
Schematic representation of the (**a**) Neratinib-loaded *Ocimum* oil nanovesicles process, and (**b**) fabrication of PVA/PVP–Propolis Dissolving Microneedles loading freeze-dried NTB-OOs (NTB-OOs/P-MNs) process. Created in BioRender. Fathy Elhabal, S. (2026) https://BioRender.com/wdas64r.

NTB-OOs were prepared by the thin-film hydration method with minor modifications. In particular, NTB (5 mg), *Ocimum basilicum* oil (100 mg),the edge activator (TPGS, Tween 80, or sodium cholate), cholesterol, and *Ocimum* oil were dissolved in a 1:10 (*v*/*v*) methanol/chloroform solution in a round-bottom flask. The organic solvent was removed under reduced pressure at 60 °C and 100 rpm using a Stuart rotary evaporator (RE300, Wolf Laboratories, North Yorkshire, UK). Complete evaporation was indicated by the formation of a thin, dry, transparent film on the flask wall. The film was then dried in a desiccator under vacuum for 2 h to remove any residual organic solvent. The film was hydrated with 10 mL of phosphate-buffered saline (PBS, pH 7.4). NTB-OOs were prepared by hydrating at 60 °C for 1 h with stirring at 100 rpm [50,51]. The vesicles were reduced in size by sonication for 10 min using a Sonix TV immersion sonicator (Model SS-series, North Charleston, SC, USA). The customized nanosuspensions of NTB-OOs are shown in Figure 1a. The nanosuspensions were stored at 4 °C overnight before further characterization.

#### 2.7.3. Physicochemical Characterization of Neratinib-Loaded Ocimum Oil Nanovesicles Formulations (NTB-OOs)

##### Determination of NTB-OOs Entrapment Efficiency Percentage (EE%)

The encapsulation efficiency (EE%) of the synthesized NTB-OOs was determined using the ultracentrifugation technique. Three milliliters were taken from each NTB-OOs and centrifuged for 1 h at 22,000 rpm and 4 °C using a refrigerated centrifuge (Sigma 3 K 30; Sigma Laborzentrifugen GmbH, Osterode am Harz, Germany). To determine the concentration of unentrapped NTB-OOs, an aliquot of the resulting supernatant was diluted with methanol to the appropriate concentration and analyzed by HPLC as described previously. The EE % was calculated by the following Equation (1) [52,53]. The study was repeated in triplicate, and the mean EE%  ±  SD was determined.

(1) $$\mathrm{EE}\%=\frac{NTB\;Total\;amount-NTB\;unentrapped\;amount}{NTB\;Total\;amount}\times 100$$

##### Particle Size, Polydispersity Index and Zeta Potential

The PS, PDI, and ZP of all synthesized NTB-OOs were measured using a Zetasizer (Malvern, Worcestershire, UK) at 25 °C. PDI is an important parameter for evaluating the size distribution of the synthesized NTB-OOs. ZP was used to evaluate the physical stability of the synthesized NTB-OOs. ZP was measured by observing mobile vesicles in an electric field. Samples of each formula were diluted to achieve sufficient light-scattering intensity before measurement. Each measurement was performed three times, and the mean ± SD was recorded [54,55].

##### Design-Expert^®^-Driven Statistical Optimization of NTB-OOs Formula

The formulation was systematically developed and optimized using Design-Expert^®^ software to achieve the desired nanosystem properties. The optimization strategy aimed to maximize EE% and ZP to ensure vesicle stability, with a strict PS in the nanometric range to ensure effective delivery. The experimental design was statistically analyzed using analysis of variance (ANOVA) to quantify the influence of input parameters on the dependent variables. Then, we applied a numerical optimization strategy in which the desirability function was the primary index, and higher values (near 1) indicated greater conformity to the optimization objectives. The best preparation, with the highest desirability value, was selected for full characterization [56,57,58].

##### Lyophilization and Sterilization of NTB-OOs Formulation

The NTB-OOs formulation was frozen at −20 °C in the presence of mannitol (2% *w*/*v*) as a cryoprotectant. Then, it was freeze-dried for 24 h at a pressure of 7 × 10^−2^ mbar and a temperature of −45 °C (Novalyphe-NL 500; Savant Instruments Corp., Midland, MI, USA) [59,60,61].

#### 2.7.4. Comprehensive Physicochemical Profiling of the Optimized Formulation (NTB-OOs)

##### Transmission Electron Microscope (TEM)

TEM (JEM-2100; JEOL Ltd., Tokyo, Japan) was used to examine the morphological structure of optimized NTB-OOs. The solution was diluted (1:100) with deionized water and stained with 2% (*w*/*v*) phosphotungstic acid. A drop of the sample was placed on a carbon-coated copper grid, and the excess liquid was blotted up with absorbent filter paper. The sample was dried at room temperature for 30 min before analysis by transmission electron microscopy [62,63].

##### Fourier Transform-Infrared Spectroscopy (FTIR)

The molecular compatibility of NTB-OOs with the carrier components and the successful incorporation of the drug into the *Ocimum* oil nanovesicles architecture were determined by Fourier Transform Infrared (FTIR) spectroscopy. FTIR spectra of NTB, NTB-OOs optimized formula, and Blank OOs were recorded using a Bruker FTIR spectrophotometer (Model 22, Coventry, UK). The preparation consisted of carefully drying the samples and then pressing them into potassium bromide (KBr) matrices to form analytical discs [64,65]. Spectral data were recorded at a fixed temperature of 25 °C in a scanning range of 4000 to 500 cm^−1^.

##### Differential Scanning Calorimetry (DSC)

Differential scanning calorimetry (DSC) was used to assess the crystallinity and potential interactions of NTB, NTB-OOs optimized formula, and Blank OOs. Samples of 4–5 mg were heated from 25 to 400 °C in flat-bottomed aluminum pans under an inert nitrogen flow of 30 mL min^−1^ at a heating rate of 10 °C min^−1^. The DSC thermogram was recorded on a Shimadzu DSC 50 differential scanning calorimeter (Shimadzu Corporation, Kyoto, Japan) calibrated with purified indium (99.9%) [66,67].

##### Stability Study of the Optimized Neratinib-Loaded Ocimum Oil Nanovesicles Formulations (NTB-OOs)

Samples of the optimized NTB-OOs were kept in a stability chamber (Remi, Mumbai, India) for 3 and 6 months as per the guidelines of the International Conference on Harmonization (ICH) under two different conditions: room temperature of 25 ± 2 °C with a relative humidity of 60 ± 5%, and accelerated condition of 40 ± 2 °C with a relative humidity of 75 ± 5%. About 100 mg of lyophilized NTB-OOs powder in sealed airtight centrifuge tubes was placed into the designated chambers. Samples were taken for three and six consecutive months. The assay and physical characteristics of the samples were assessed at each time interval [68,69,70]. To evaluate physical stability, pre-storage and post-storage values of EE%, PS, PDI, and ZP were compared. Each stability sample was tested in triplicate.

#### 2.7.5. Fabrication of PVA/PVP–Propolis Dissolving Microneedles Loading Freeze-Dried NTB-OOs (NTB-OOs/P-MNs)

Microneedle tips for dissolution were prepared using different ratios of polyvinyl alcohol (PVA), polyvinyl pyrrolidone K90 (PVP K90), propolis, and freeze-dried neratinib-loaded *Ocimum* oil vesicles (NTB-OOs), as shown in Table 3. The specified amounts of PVA and PVP K90 were dissolved in distilled water under constant stirring until clear, homogeneous polymeric solutions were obtained. Propolis was then added to the polymeric blend at concentrations of 0.5, 1.0, and 1.5% *w*/*w*, and the mixture was stirred gently to ensure uniform distribution within the PVA/PVP matrix. Next, a known amount of freeze-dried NTB-OOs was added to the prepared PVA/PVP–propolis solution, and the mixture was stirred gently until a homogeneous vesicle-loaded microneedle-tip formulation was obtained. A known volume of the NTB-OOs/PVA/PVP–propolis tip solution was gently dispensed into a 1 cm × 1 cm polydimethylsiloxane (PDMS) microneedle mold containing 100 conical microneedle cavities arranged in a 10 × 10 array. A vacuum was applied for 20 min to ensure complete filling of the microneedle cavities and to remove any trapped air bubbles [36,71]. After the cavities were filled, a drug-free backing layer of a PVA/PVP polymeric solution was applied to the microneedle tips to increase mechanical strength and ease handling of the final patches. The filled molds were then dried overnight at room temperature, protected from light. After careful demolding, the synthesized NTB-OOs/P-MNs were stored in desiccators for further characterization [72].

#### 2.7.6. Physical Characteristics of PVA/PVP–Propolis Dissolving Microneedles Loading Freeze-Dried Neratinib-Loaded Ocimum Oil Vesicles (NTB-OOs/P-MNs)

##### Mechanical Strength and Penetration Capability Test

The mechanical strength and skin-penetrating ability of NTB-OOs/P-MNs were evaluated using the Parafilm^®^M penetration model. Parafilm^®^M was stacked in eight layers, each about 0.13 mm thick, to a total thickness of almost 1 mm. The Parafilm^®^ sheets were stacked, placed on a flat surface, and kept at 25 °C before testing. Parafilm^®^M was chosen as a model synthetic skin due to its flexibility and suitability for the preliminary assessment of microneedle penetration efficiency. The microneedle arrays (n = 3) were placed vertically on the Parafilm^®^M stack and compressed for 30 s at a constant force of 32 N (~3.2 kgf) with a custom-made compression device. The pressure was applied to replicate the pressure exerted by the thumb when microneedles are inserted. After insertion, the microneedle arrays were carefully removed, and the layers of Parafilm^®^M were sequentially peeled off [32,73]. The penetration efficacy rate was calculated as the ratio of observable holes to the total number of microneedles in each array. The reduction in microneedle height was quantified to evaluate their durability against deformation or breakage during insertion. The depth of penetration was determined by the deepest layer of Parafilm^®^M that exhibited visible perforations and by the thickness of each layer. The percentage decrease in height, penetration rate, and projected penetration depth were calculated using the following equations (Equations (2) and (3)). The mechanical adequacy of the microneedle arrays was determined by the reduction in height after application, which should be less than 20%, and by the fact that more than 80% of the microneedles penetrated four to six layers of Parafilm^®^M, indicating sufficient mechanical integrity and insertion efficacy for dermal application [31,74].

(2) $$\%\mathrm{Height}\;\mathrm{Reduction}=\;\frac{Initial\;height-Final\;heigh}{Initial\;height}\;\times 100\%$$

(3) $$\%\mathrm{Penetration}\;\mathrm{capability}=\frac{Number\;of\;holes}{Total\;needles}\;\times 100\%$$

These parameters help determine the microneedles’ structural integrity under applied force and insertion efficacy into skin-like substrates.

##### Determination of Drug Content

The homogeneity of NTB incorporation in microneedle arrays was assessed by quantification of the drug content of the NTB-OOs/P-MNs. Three different microneedle arrays were accurately weighed and then completely dissolved in 10 mL of methanol-buffer solution (50:50 *v*/*v*, pH 5.5). The samples were stirred at 500 rpm for 24 h at 25 °C to ensure complete dissolution of the polymeric matrix and the complete extraction of NTB from the microneedle arrays. The solutions obtained after extraction were filtered through a 0.45 μm PVDF membrane filter and analyzed using a validated HPLC method, as previously described [30,75]. The drug content was expressed as % of recovery with respect to theoretical drug loading and calculated according to the following Equation (4):

Drug content (%) = Actual drug content/Theoretical drug content × 100 (4)

Results were expressed as mean ± standard deviation. A high drug-content percentage indicated efficient integration and uniform distribution of NTB-OOs within the NTB-OOs/P-MNs microneedle matrix.

##### Dissolution Time Assessment

To mimic in vivo dissolution, microneedles were administered after euthanasia, and full-thickness mouse skin was excised; hair was shaved off with an electric clipper, subcutaneous fat was carefully cleaned, and skin was stored in phosphate-buffered saline at −20 °C until use (within one week). MNs were inserted into the epidermis using manual thumb pressure, and extended adhesion was mimicked by placing a 5 g flat weight on top of the P-MNs. Microneedle patches were systematically withdrawn after the designated time intervals of 2, 4, 6, and 8 min. The morphology of the extracted microneedles was observed under light microscopy, enabling the quantification of dissolution at the needle tip. This approach allows the determination of dissolution kinetics and release rate, which is important for time-controlled release applications [76,77].

##### Water Loss During Drying (LOD) Test

The loss on drying of the microneedle arrays was evaluated gravimetrically in accordance with the United States Pharmacopeia (USP). To summarize, individual microneedle arrays (n = 3) with an initial mass of ca. 20 mg were accurately weighed after drying. Specimens were dried in a vacuum oven at 105 °C for 3 h or until a constant weight was reached. The microneedle arrays were allowed to cool in a desiccator before being reweighed, and the final dried weight was recorded [33,78,79]. The percentage of LOD was calculated from the following equation (Equation (5)):

(5) $$\%\mathrm{LOD}\;=\;\frac{Initial\;weight-Final\;weight}{Initial\;weight}\;\times 100\%$$

A lower LOD (<10%) is generally preferred to ensure mechanical stability, prevent microbial growth, and increase shelf life.

#### 2.7.7. Characterization of Optimized PVA/PVP–Propolis Dissolving Microneedles

##### Scanning Electron Microscopy (SEM)

SEM (JXA-840; JEOL, Japan) was used to examine the surface morphology of the NTB-OOs/P-MNs formulation. The examination was conducted after coating the NTB-OOs/P-MNs formulation with gold under vacuum [80].

##### Fourier Transform Infrared (FTIR) Spectroscopy

An FTIR spectrometer was used to record room-temperature spectra of blank P-MNs, NTB-OOs/P-MNs, and their constituent parts between 4000 and 500 cm^−1^ (model 22, Bruker, Coventry, UK), as previously described [81].

##### Differential Scanning Calorimetry (DSC)

The thermal properties, crystallinity, and interactions of the blank P-MNs, NTB-OOs/P-MNs, and their constituent parts were examined using differential scanning calorimetry (DSC-60; Shimadzu, Kyoto, Japan) at temperatures from 25 to 350 °C under nitrogen purge at 100 mL/min, as described previously [82,83].

#### 2.7.8. Drug Release Studies

##### Drug Release and Kinetic Modeling

The drug release was studied using the dialysis membrane method; three dialysis bags were prepared, each containing 5 mL of the optimized NTB-OOs formulation (equivalent to 5 mg NTB). Equal volumes (5 mL) of NTB suspension containing 5 mg of NTB and NTB-OOs/P-MNs were placed in separate dialysis bags for direct comparison. For each dissolution medium, all formulations were tested: each formulation and the drug suspension were transferred into individual dialysis bags and then placed in 200 mL of physiological-pH phosphate buffer (PB, pH 7.4). Tween 80 (0.5% *v*/*v*) was added to PBS (pH 7.4) to maintain sink conditions. Samples were collected at 0.5, 1, 2, 3, 4, 5, 6, 12, 24, and 48 h intervals. At each time interval, 4 mL samples were collected and replaced with fresh medium to maintain uniform conditions. The same procedure was used to determine the release profile of an NTB suspension against the NTB-OOs and NTB-OOs/P-MNs formulations. The prepared aliquots were filtered through a 0.45 μm Millipore filter, and the amount of released drug was determined by HPLC (Phenomenex, Torrance, CA, USA) as described previously. The cumulative percentage of NTB released was calculated and plotted versus time. The dissolution profiles were analyzed using several mathematical kinetic models to elucidate the transport phenomena governing NTB release from different formulations. The experimental results were analyzed with zero-order, first-order, Higuchi, and Korsmeyer–Peppas models. The correlation coefficient of each model was explored. The model with the highest R^2^ was considered the representative mechanism [84,85,86].

##### Ex Vivo Permeation Study

Ex vivo permeation of NTB from NTB suspension, NTB-OOs, and NTB-OOs/P-MNs was evaluated using vertical Franz diffusion cells. Excised rat skin was positioned between the donor and receptor compartments, with the stratum corneum facing the donor side. The receptor compartment contained phosphate buffer (pH 7.4) with Tween 80 (0.5% *v*/*v*) and was maintained at 32 ± 0.5 °C with constant stirring. The respective formulation was added to the donor compartment for each NTB dosage [50,87,88]. Samples were collected from the receptor compartment at fixed time intervals and immediately replaced with fresh receptor medium. The total penetration per unit area, steady-state flux, permeability coefficient, and enhancement ratio were determined by the HPLC method described above. All experimental procedures were performed in triplicate.

### 2.8. In Vitro Cytotoxicity Study Using Human Breast Adenocarcinoma Cell Line (MCF-7)

The human breast adenocarcinoma cell line MCF-7 was purchased from the American Type Culture Collection (ATCC, USA). Cells were grown in Dulbecco’s Modified Eagle’s Medium (DMEM) supplemented with 10% fetal bovine serum (FBS) and 1% penicillin-streptomycin at 37 °C in a humidified incubator with 5% CO_2_. Cells were seeded in 96-well plates (1.0 × 10^3^ cells/well) and treated with different concentrations of doxorubicin (DOX), NTB, or NTB-OOs for 48 h. Cell viability was determined by the MTT (3-(4,5-dimethylthiazol-2-yl)-2,5-diphenyltetrazolium bromide) assay [2,89,90]. Cell viability percentage (CV%) was calculated as Equation (6):

(6) $$\mathrm{CV}\%\;=\;\frac{Absorbance\;of\;treated\;cells}{Absorbance\;of\;controled\;cells}\;\times 100$$

Dose–response curves and IC_50_ values were calculated by using GraphPad Prism version 10.

### 2.9. In Vivo Study

#### 2.9.1. Induction of the Solid Form of Ehrlich Carcinoma (ESC) and Experimental Design

Female Swiss albino mice were selected according to the criteria of the Solid Ehrlich carcinoma (SEC) model. The Ehrlich ascites carcinoma (EAC) cell line was kindly provided by the Unit of Pharmacology and Experimental Oncology, National Cancer Institute, Cairo University, Egypt. EAC cells were appropriately diluted (2 × 10^6^) and transplanted into female Swiss albino mice by serial subcutaneous injections in the left mammary fat pad. Tumor volume was measured on the 14th day after inoculation, reaching about 100 mm^3^. Mice were sacrificed, and cells were isolated from the ascitic fluid using a syringe and diluted with normal saline at a 1:1 ratio [2,91]. Viable cells were then inoculated subcutaneously (0.2 mL/mouse) into the right thigh of the left mammary fat pad of mice [92]. ESC mice formed tumor masses at 14 days; 40 mice bearing ESC tumors were randomly divided into four groups of 10 mice each (n = 10/group).

**Figure 2:**
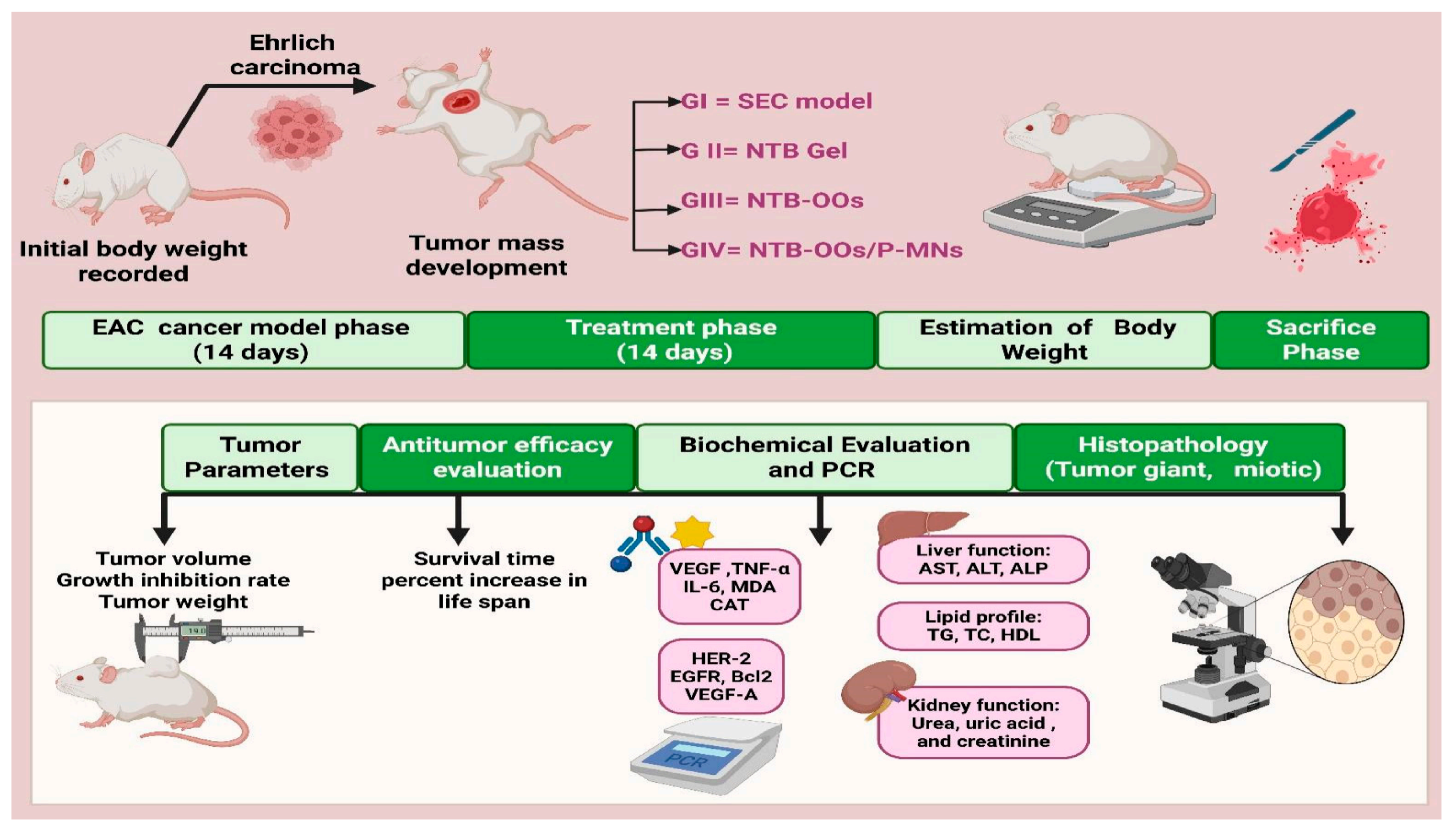
Schematic representation of the in vivo study of the Ehrlich carcinoma (ESC) model. Created in BioRender. Fathy Elhabal, S. (2026) https://BioRender.com/kvu37ex.

Group I (ESC model group, untreated) and three treated groups: Group II (treated with NTB incorporated in gel containing 2% (*w*/*w*) HPMC), Group III (treated with NTB-OOs incorporated in gel containing 2% (*w*/*w*) HPMC), and Group IV (treated with NTB-OOs/P-MNs). Figure 2 demonstrates that all treatments were initiated on day 14 after tumor induction and administered transdermally every other day for two weeks.

#### 2.9.2. Assessment of Tumor Volume, Growth Inhibition Rate, and Tumor Weight

Mice were observed daily; tumor growth was assessed using two-dimensional measurements with Vernier calipers, taken every 3 days. Ellipsoidal tumor volume (V) was quantified and calculated using the following Formula (7) [1,93,94]:

V = (D × d^2^)/2 (7)

where D is the longest diameter and d is the shortest diameter.

Tumor growth inhibition rate (TIR%) was calculated as (C − T)/C × 100(8)

where T is the mean tumor volume in the treatment group, and C is the mean tumor volume in the control group. The weight of the entire tumor mass was measured at the end of the experiment, after removal of the mass from the thigh [95,96].

#### 2.9.3. Determination of the Survival Time, Percent Increase in Life Span

The animals were monitored daily for mortality until the termination of the experiment. The experiment was ended by the unexpected death of the animals, and the mean survival time (MST) was calculated using the following Equation (9) [97]

MST  =  (day of first death  +  day of last death)/2(9)

The percentage increase in life span (ILS) was calculated using the Nicol and Prasad Formula (10).

ILS% = (T − C)/C × 100(10)

where T is the MST of the treated animals, and C is the MST of the control group. T/C% (treated vs. Ehrlich control) was calculated by dividing the mean survival time (MST) of the animals treated by the MST of the control group. The National Cancer Institute (NCI) defines a drug as having significant antitumor activity when T/C is greater than 125%, and ILS is greater than 25% [97].

#### 2.9.4. Biochemical Evaluation of Tumor Tissue Biomarkers

Biochemical effects of different therapies were assessed in excised tumor tissues by determining apoptotic, angiogenic, inflammatory, oxidative stress, and antioxidant biomarkers. Tumor-bearing animals were divided into four groups: untreated Ehrlich solid carcinoma control group (ESC), NTB, NTB-OOs, and NTB-OOs/Propolis-MNs. The animals were sacrificed at the end of the treatment period, and the tumor tissues were carefully removed, washed with cold normal saline, dried, weighed, and homogenized in cold phosphate buffer solution. Homogenization of tumor tissue from three different animals in each group was performed individually and analyzed separately. Each animal was treated as a biological replicate (n = 3 biological replicates per group). When technical replicate measurements were performed, these were averaged to produce a single value for each animal prior to statistical evaluation. Caspase-3, a marker of apoptosis, was used to evaluate treatment-induced cell death in tumor tissues. Vascular endothelial growth factor (VEGF) was assayed as a marker of angiogenesis, and tumor necrosis factor-α (TNF-α) and interleukin-6 (IL-6) as inflammatory mediators. We identified troponin-1 as a biomarker linked to tissue damage. Also, malondialdehyde (MDA), an index of lipid peroxidation and oxidative stress, and catalase (CAT) activity, a measure of intrinsic antioxidant defense, were estimated [98,99]. All biomarkers were measured by commercially available sandwich ELISA kits (LifeSpan Biosciences, Inc., USA) according to the manufacturer’s instructions. Standards and samples were added to the microplate pre-coated wells and incubated with biotinylated detection antibodies and streptavidin-HRP conjugate. Color development was performed using TMB substrate, and the reaction was stopped with 2N H_2_SO_4_. Absorbance was measured at 450 nm using a microplate reader, and cytokine or protein concentrations were calculated from the respective standard curves and normalized to tissue weight (pg/g tissue) [59].

#### 2.9.5. Liver Function Tests (LFTs), Kidney Function Tests and Lipid Profile Assessment

Liver function (aspartate aminotransferase activity), renal function (urea, uric acid, creatinine) and total bilirubin were assessed. Alanine transaminase and alkaline phosphatase activity were determined using commercial reagent kits and a spectrophotometer. Triglyceride (TG) concentrations in female mouse plasma, total cholesterol (TC) levels, and high-density lipoproteins (HDL) were assayed [100,101,102].

#### 2.9.6. Quantitative Reverse Transcription PCR (RT-qPCR)

Total RNA was isolated from homogenized tissues of different experimental groups using QIAzol reagent (Qiagen, Germany) following the manufacturer’s instructions. The RNA concentration and purity were measured using the NanoDrop spectrophotometer (Thermo Fisher Scientific, USA). Total RNA (1 μg) was reverse transcribed to complementary DNA (cDNA) using the SensiFAST™ cDNA Synthesis Kit (Bioline, London, UK) as recommended by the manufacturer. The resulting cDNA was amplified using a real-time PCR machine Applied Biosystems 7500 real-time PCR system (Thermo Fisher Scientific, Waltham, MA, USA) under the following cycling conditions: 2 min at 95 °C for initial denaturation, followed by 40 cycles of 10 s at 95 °C and 30 s at 60 °C. Each 20 μL reaction contained 10 μL HERA SYBR Green PCR Master Mix (Willowfort, Nottingham, UK), 2 μL cDNA template, 1 μL forward primer, 1 μL reverse primer and 6 μL nuclease-free waters. Gene expression analysis of HER2, EGFR, VEFG-A and Bcl_2_ was performed by the 2^−ΔΔCt^ method using GAPDH as an internal control gene. Primer specificity was validated using Primer-BLAST and product specificity was confirmed using melting-curve analysis [1,103]. The primers employed in this study are listed in Table 4. Primers were designed using Primer3 (version 4.1.0; https://primer3.ut.ee/ (accessed on 24 May 2026)). Primer specificity was checked using Primer-BLAST (https://www.ncbi.nlm.nih.gov/tools/primer-blast/ (accessed on 24 May 2026)), and melting-curve analysis was performed.

### 2.10. Histopathologic Analysis

Tumor tissue samples for histopathological investigations were immediately immersed in 10% buffered formalin solution. The fixed tissues were embedded in paraffin wax and sectioned at an average thickness of 5 µm using a microtome. The sections were then stained with hematoxylin and eosin stains. Stained slices were photographed and examined under a light microscope [2,5].

### 2.11. Statistical Analysis

Data are presented as mean ± SD, with n representing independent biological replicates unless otherwise noted. We used a one-way ANOVA with Tukey’s multiple-comparisons test to compare multiple groups for single-time-point outcomes. Longitudinal tumor volume data were analyzed with two-way repeated-measures ANOVA with treatment and time as factors, followed by appropriate multiple-comparisons testing. Normality and homogeneity of variance were tested prior to the parametric analysis. *p* < 0.05 was deemed as statistically significant. Effect sizes were also reported as η^2^ for one-way ANOVA and partial η^2^ for repeated-measures analyses, along with 95% confidence intervals for primary between-group comparisons. The levels of statistical significance were *p* > 0.05; * (*p* < 0.05); ** (*p* < 0.01); *** (*p* < 0.001); and **** (*p* < 0.0001).

## 3. Results and Discussion

### 3.1. GC/MS Analysis of the Extracted Ocimum basilicum Oil

**Figure 3:**
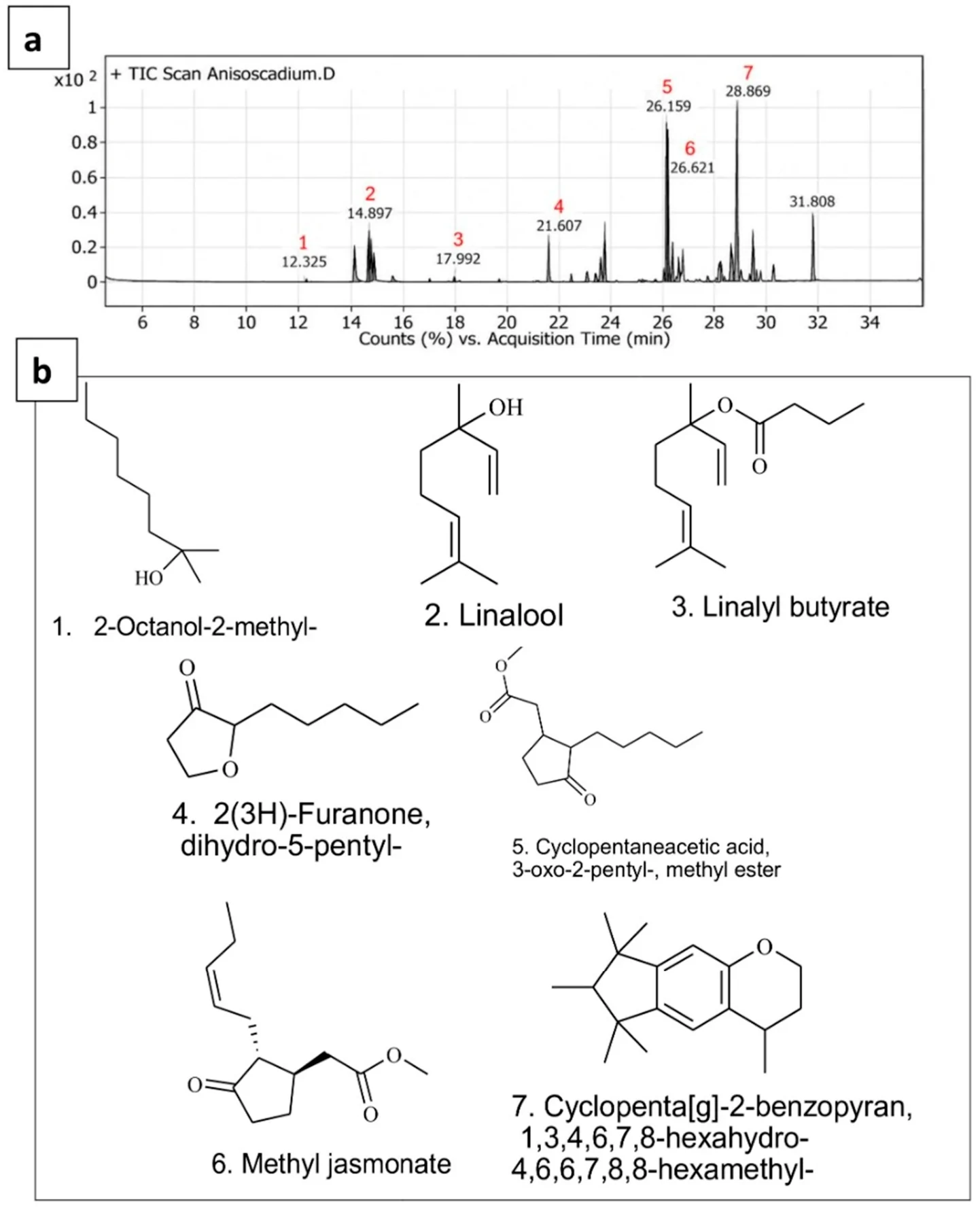
(**a**) GC/MS chromatogram of *Ocimum basilicum* essential oil. (**b**) Structure of the compounds identified from GC/MS analysis of the *Ocimum basilicum* essential oil.

GC/MS analysis of *Ocimum basilicum* oil revealed seven compounds with retention times ranging from 12.325 to 28.869 min, as shown in Figure 3 and Table 5. Identified compounds were alcohol, esters, lactone derivatives, oxygenated terpenoid-related compounds, and jasmonate derivatives. Among the identified compounds, linalool was one of the major constituents, representing 10.12% of the total chromatographic area (Rt = 14.897 min). Linalool is a well-known oxygenated monoterpene widely distributed in *Ocimum* species and is recognized for its antimicrobial, antioxidant, anti-inflammatory, sedative, and insecticidal activities. The relatively high abundance of linalool is consistent with previous reports identifying it as a characteristic constituent of *O. basilicum* essential oil, although its concentration may vary by cultivar, geographical origin, harvesting season, and extraction method. The detection of methyl jasmonate (Rt = 26.621 min, 12.89%) further highlights the oil’s biochemical complexity. Methyl jasmonate is a naturally occurring jasmonate derivative involved in plant defense signaling and stress responses. In addition to its physiological role in plants, methyl jasmonate has attracted considerable pharmacological interest because of its reported antioxidant, anti-inflammatory, antimicrobial, and anticancer activities. Therefore, its relatively high proportion in the analyzed oil may enhance its potential therapeutic and industrial applications. The chromatogram also showed a significant proportion of 2(3H)-furanone, dihydro-5-pentyl- (Rt = 21.607 min; 10.92%), a lactone compound associated with flavor characteristics and potential antimicrobial activity. Similarly, linalyl butyrate (Rt = 17.992 min; 2.12%) was identified as a minor oxygenated monoterpene ester that contributes to the pleasant floral aroma of basil and possesses potential biological activities. A minor constituent, 2-Octanol, 2-methyl- (Rt = 12.325 min; 1.14%), was also detected. Although present in low concentration, branched-chain alcohols can contribute to the overall aroma profile and may exhibit mild antimicrobial effects. The analysis additionally identified Cyclopentaneacetic acid, 3-oxo-2-pentyl-, methyl ester (Rt = 26.159 min) and Cyclopenta[g]-2-benzopyran, 1,3,4,6,7,8-hexahydro-4,6,6,7,8,8-hexamethyl- (Rt = 28.869 min). These compounds belong to oxygenated cyclic derivatives that have been reported in several plant extracts and may contribute to antioxidant and biological activities. Overall, the GC–MS profile demonstrates that the oil of *O. basilicum* is rich in oxygenated compounds, particularly oxygenated monoterpenes and jasmonate derivatives, which are known to possess diverse pharmacological activities. The predominance of linalool together with methyl jasmonate and other oxygenated metabolites may explain the antioxidant, antimicrobial, anti-inflammatory, and therapeutic properties for *O. basilicum* [104,105]. The lipophilic constituents present in high abundance in the oil may also favor the successful development of lipid-based nanoformulations such as nanoemulsions, nanovesicles, nanophytosomes, solid lipid nanoparticles, or nanostructured lipid carriers. These compounds can be used as the active oil phase of the nanosystem. Surfactants, phospholipids, or lipid carriers facilitate a reduction in particle size, enhance dispersion in aqueous media, improve physical stability, and protect the active components from degradation. This is significant because numerous components of essential oils exhibit low water solubility, volatility, chemical instability, and limited bioavailability. A nanoformulation exhibiting enhanced solubility, stability, cellular absorption, regulated release, and direct interaction with cancer cells can surmount these constraints. From a pharmacological standpoint, linalool and methyl jasmonate may be among the most significant compounds that enhance the extract’s potential anticancer efficacy. Linalool has been reported to possess several anticancer properties, including inhibition of cell proliferation, modulation of oxidative stress and the cell cycle, mitochondrial dysfunction, and induction of apoptosis in various cancer cell models. Moreover, methyl jasmonate, a plant-derived jasmonate, has been documented to possess anticancer properties through mitochondrial impairment, disruption of cancer cell energy metabolism, production of reactive oxygen species, and promotion of apoptosis. Thus, the substances found in greater quantities may have a double function [24,106]. First, their lipophilic nature facilitates easy loading in lipid-based nanocarriers, improving the formation and stability of the nanoformulation. Secondly, the biological functions they documented, particularly those of linalool and methyl jasmonate, could enhance the anticancer efficacy of the nanoformulated oil relative to the unencapsulated oil. Consequently, the nanoformulation of *Ocimum* oil may represent a viable strategy to enhance the bioavailability, stability, delivery, and therapeutic effectiveness of its bioactive components. However, this anticancer potential needs to be validated experimentally by comparing the free oil and the nanoformulated oil using in vitro cytotoxicity assays, apoptosis markers, cell-cycle analysis, and cellular uptake studies [23,107].

### 3.2. Evaluation of NTB-OOs Formulations

#### 3.2.1. Entrapment Efficiency (EE%)

**Figure 4:**
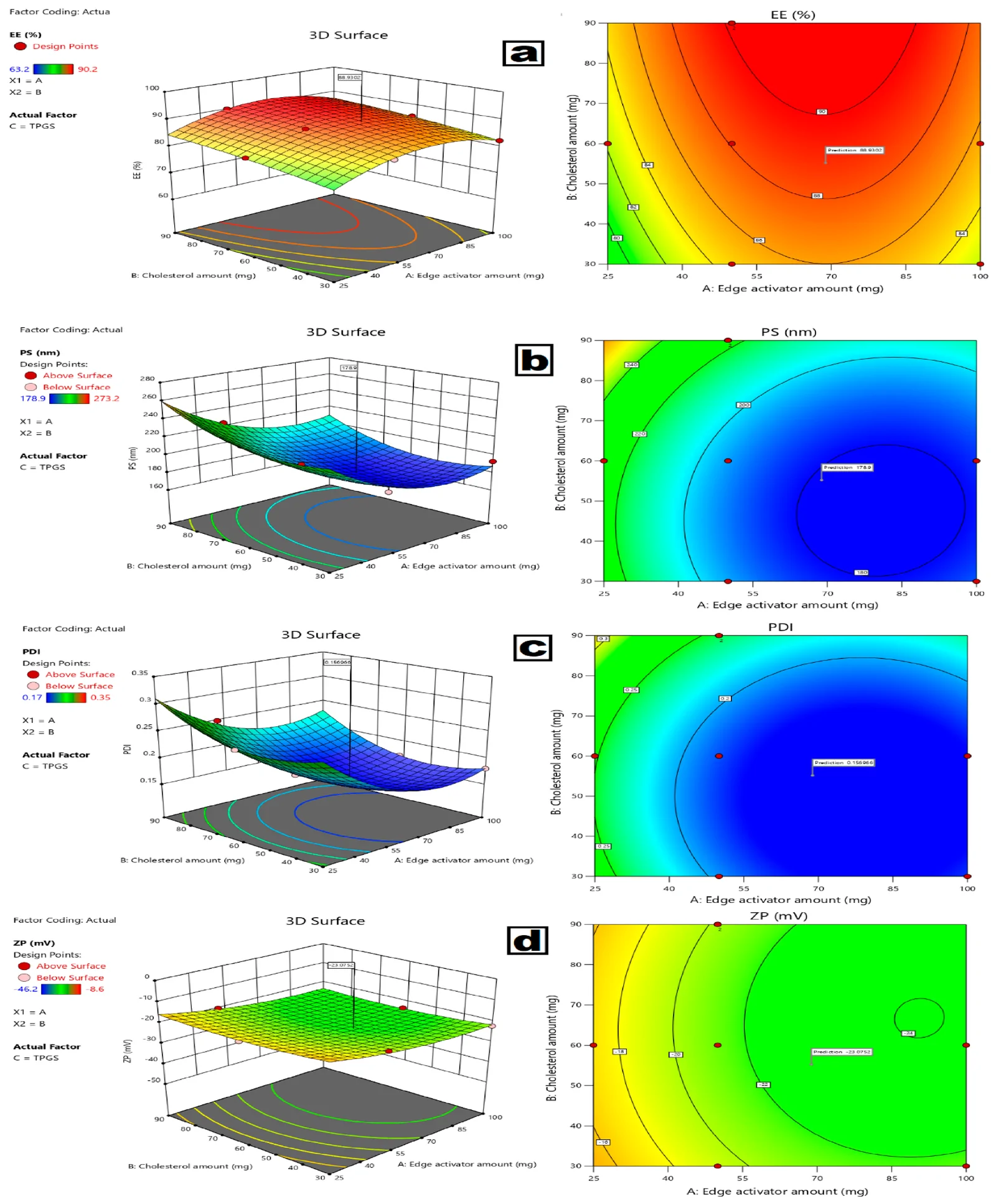
3D Response surface plot showing effect of (A) Edge activator amount, (B) Cholesterol amount, (C) Edge activator types on (Y1) Entrapment efficiency percentage (EE%) (**a**), (Y2) Particle size (PS) (**b**), (Y3) polydispersity index (PDI) (**c**), and (Y4) zeta potential (ZP) (**d**).

Entrapment efficiency is one of the key parameters used to characterize drug incorporation into vesicular nanocarriers; however, it should be interpreted together with the drug-to-carrier loading ratio, as it reflects the formulation’s ability to entrap and protect the drug within the vesicular structure. The efficient encapsulation of drugs with poor water solubility, such as NTB, is very important, as it can improve drug delivery to the site of action, inhibit degradation, and enable solubilization. The EE% values of the synthesized NTB-OOs ranged from 63.2 ± 0.91% to 90.2 ± 0.94%, as shown in Table 2. The EE % was highest in run 6 (50 mg edge activator, 90 mg cholesterol, and TPGS) and lowest in run 18 (100 mg edge activator, 30 mg cholesterol, and sodium cholate). Thus, the EE% was significantly affected by cholesterol concentration and the type of edge activator. Among the edge activator formulations studied, those with TPGS showed higher encapsulation efficiency rates than those with sodium cholate and Tween 80. This phenomenon can be attributed to the amphiphilic configuration of TPGS acting as a stabilizer, emulsifier, and solubilizing agent in the vesicular bilayer [108,109]. TPGS is a pharmaceutical excipient that is widely used and may enhance solubilization, drug delivery and permeation, and overcome P-glycoprotein-mediated drug resistance. The lipophilic drug and oil phase may be constituted by the hydrophobic vitamin E moiety, while the hydrophilic PEG chain provides steric stabilization and dispersion. This dual functionality could result in improved NTB retention in the vesicular matrix and reduced drug efflux. EE% also tends to increase with high cholesterol levels. This could be linked to cholesterol’s ability to strengthen the vesicular bilayers, reduce permeability, increase membrane rigidity, and prevent drug leakage. Cholesterol is often incorporated into vesicular systems to improve bilayer packing and to stabilize vesicles [110,111]. The results of the present study showed that the formulations, especially those containing TPGS at high concentrations of cholesterol, achieved high entrapment values. Thus, cholesterol created a more lipophilic and rigid environment that favored the retention of NTB in the bilayer. Formulations with sodium cholate showed a reduction in EE%, with the effect more pronounced at higher concentrations of the edge activator. This might be due to the ionic and bile-salt properties of sodium cholate, which can increase bilayer fluidity and membrane permeability. The vesicular bilayer can be disturbed by high concentrations of edge activators, leading to defects or pore formation that cause partial drug leakage and lower the EE, as shown in Figure 4a. Edge activators are essential for increasing vesicle flexibility, but their concentration must be controlled to avoid destabilizing the bilayer.

#### 3.2.2. Particle Size, Polydispersity Index and Zeta Potential

##### Particle Size (P.S.)

Particle size is a key characteristic of nanovesicular drug delivery systems because it affects physical stability, drug release, cellular uptake, tissue penetration and biological efficacy. Nanocarriers are especially advantageous for poorly water-soluble pharmaceuticals because they enhance the apparent solubility, dissolution rates, and bioavailability. The particle size of NTB-OOs was in the range of 172.8 ± 0.57 to 273.2 ± 0.88 nm, as shown in Table 3, which indicates the successful preparation of nanosized vesicles. The smallest particle size was obtained in run 20 with the combination of 100 mg of edge activator, 30 mg of cholesterol and TPGS. On the other hand, the highest particle size (273.2 ± 0.88 nm) was observed in run 15 which had 25 mg edge activator, 90 mg cholesterol and sodium cholate. This observation may be related to the effects of surfactant concentration and cholesterol content on the bilayer organization and vesicle size reported in previous studies [112]. In general, the quantity of the edge activator was found to reduce particle size. However, this might be due to the surface activity of edge activators, which can decrease the interfacial tension during vesicle formation, facilitate lipid dispersion and encourage vesicle curvature. At the lower edge activator concentrations, it could happen that there are not enough surfactants to cover new vesicle surfaces, which may result in the formation of larger particles and aggregation. In contrast, the high concentration of edge activators could lead to the formation of smaller, more flexible vesicles due to increased bilayer flexibility and distribution. The particle size was also affected by the type of edge activator. In general, formulations with TPGS exhibited smaller vesicles than those with Tween 80. Tween 80 formulations exhibited relatively bigger particle sizes, which could be attributed to its bulky nonionic structure and its effect on bilayer organization. Earlier work on other edge activators has shown that Tween 80 can generate larger vesicles and reduce zeta potential compared with bile salt-based edge activators, such as sodium cholate [113]. The cholesterol content tended to increase particle size, especially at high levels. This is because the presence of cholesterol can increase the bilayer’s rigidity and thickness, promoting the formation of larger vesicles. The higher lipid content might either promote vesicle aggregation or increase the hydrophobic volume of the bilayer. Thus, while cholesterol enhanced the drug entrapment, excess cholesterol may increase the vesicle size. Therefore, the optimized formulation contained a moderate cholesterol content of 55 mg, rather than the maximum level, as shown in Figure 4b.

##### Polydispersity Index (PDI)

PDI is a measure of particle-size distribution and formulation uniformity. Lower PDI values indicate a more uniform population of vesicles whereas higher PDI values indicate a wider size distribution and possible aggregation. The polydispersity index (PDI) and particle size of lipid nanocarrier systems play significant roles in stability, reproducibility, and clinical efficacy. The PDI values of the NTB-OOs were 0.17 ± 0.07 to 0.35 ± 0.05 (Table 2). Most of the formulations exhibited PDI values around 0.30 or less, indicating a relatively narrow size distribution and good homogeneity. The optimized formulation (run 16) exhibited the minimum PDI, which indicated that the TPGS produced highly uniform vesicular dispersions. The optimized formulation showed the highest PDI value of 0.16 ± 0.01, indicating improved uniformity. In general, higher concentrations of the edge activator resulted in lower PDI values. This is due to increased surfactant surface coverage on the vesicles, which reduces aggregation and promotes the formation of uniformly dispersed nanovesicles. This is consistent with the particle-size results and suggests that vesicles were more uniform and smaller in size as the edge-activator concentration increased. Increased PDI was found in high cholesterol, especially with low levels of the edge activator [114,115]. This may be attributed to increased vesicle rigidity and a greater tendency to aggregate in the presence of high lipid content but insufficient surfactant coverage. Hence, the correct ratio of cholesterol to edge activator is crucial for generating uniform vesicles. In the optimized formulation, this balance was achieved by the inclusion of a moderate amount of cholesterol and TPGS as edge activator, as shown in Figure 4c.

##### Zeta Potential (Z.P.)

Zeta potential is an indicator of the surface charge of nanoparticles and an important measure of colloidal stability. The absolute values of zeta potential are increased, regardless of polarity, leading to electrostatic repulsion between particles and a decrease in aggregation. Zeta potential values of ±30 mV or higher are generally considered more stable, whereas lower absolute values may require steric stabilization with surfactants or polymers. The zeta potential measurements for NTB-OOs varied between −8.6 ± 0.35 and −46.2 ± 0.34 mV, as illustrated in Table 2. All formulations showed a negative surface charge, attributable to the presence of negatively charged or polar groups on the vesicles’ surface. This was more pronounced in the formulations containing sodium cholate. Run 11 exhibited the lowest zeta potential values among formulations containing sodium cholate, reaching −46.2 ± 0.34 mV. Na-cholate is an anionic bile salt and can contribute a large negative charge to the surface of the vesicle, which is to be expected. The TPGS-containing formulations had moderate negative zeta potential values ranging from −17.5 to −23.5 mV. While these are lower in magnitude than sodium cholate formulations, TPGS PEG chains can provide steric stabilization that can reduce aggregation even without significant electrostatic repulsion. This explains the good particle size and PDI values obtained for TPGS formulations despite moderate zeta potential values. TPGS is used as a stabilizing and solubilizing excipient in pharmaceutical delivery systems. TPGS with a PEGylated structure improved dispersion stability. Tween 80 formulations had the lowest negative zeta potential values, ranging between −8.6 and −14.8 mV. The reduced values are attributed to the nonionic nature of Tween 80, which does not produce a significant surface charge. Hence, Tween 80 formulations had a comparatively larger particle size and a higher PDI than TPGS formulations [116,117]. Although sodium cholate yielded the highest absolute zeta potential, the optimum formulation was not achieved due to lower EE% and, in some cases, larger particle size and higher PDI. Thus, the optimized formulation was not characterized by the most negative zeta potential but by an optimal balance among high EE%, nanosized particles, minimal PDI, and a satisfactory zeta potential, as shown in Figure 4d. The results indicated that the physicochemical properties of NTB-OOs were influenced by the combined effects of the amounts of edge activator and cholesterol, and the type of edge activator. TPGS was found to be the most suitable edge activator as it has good entrapment efficiency, smaller particle size, low polydispersity index, and appropriate zeta potential. Cholesterol improved drug entrapment by stabilizing and rigidifying the vesicular bilayer. But excess cholesterol increased the size and polydispersity index (PDI) of the particles. On the other hand, an increase in the amount of edge activators reduced the particle size and improved the size uniformity. However, excess edge activators could affect the drug entrapment due to increasing the permeability of the bilayers. The optimized formulation containing 68 mg of edge activator, 55 mg of cholesterol, and TPGS showed a zeta potential of −23.1 ± 0.35 mV, a polydispersity index (PDI) of 0.16 ± 0.01, a particle size of 198.9 ± 0.98 nm, and an encapsulation efficiency (EE%) of 88.9 ± 0.45%. The results showed that a stable, homogeneous and effective vesicular system of NTB loaded with *Ocimum* oil was successfully formulated. High EE% suggests efficient NTB loading, and low PDI indicates uniformity. Negative zeta potential and TPGS steric stabilization may improve dispersion stability. Moreover, the small particle size may increase drug dissolution and cellular uptake. Thus, NTB-OOs can serve as a nanovesicular platform for improved NTB delivery. The natural oil phase of *Ocimum* oil can be used to improve the formulation by solubilizing the lipophilic drug and providing bioactive phytoconstituents. However, further studies are required to confirm the therapeutic efficacy of the optimized NTB-OOs formulation, including in vitro release, stability, cytotoxicity, cellular uptake, apoptosis and in vivo pharmacokinetic studies.

#### 3.2.3. Optimization of NTB-OOs Formulations

The quadratic model was used to evaluate the encapsulation efficiency (EE%), particle size, polydispersity index (PDI), and zeta potential of NTB-OOs nanovesicles to increase the quantities and varieties of cholesterol and edge activator. For all responses, the quadratic model was selected, indicating a non-linear relationship between the formulation variables and the observed responses, with interaction effects and curvature. The dimensions, uniformity, surface charge, and drug encapsulation of vesicular systems can be significantly impacted by minor modifications to the formulation of surfactants and lipids. The results of the ANOVA tests show that all models are statistically significant with high F values and low *p*-values (*p* < 0.0001) for each response. The selected independent variables significantly affected the physicochemical properties of NTB-OOs, as shown in Table 6. The EE% model exhibited a strong capacity to explain the changes in entrapment efficiency (F-value = 485.00, R^2^ = 0.998). The adjusted R^2^ of 0.996 and predicted R^2^ of 0.985 indicated a good agreement between the experimental and predicted values. The particle size model was statistically significant with F = 78.34 and R^2^ = 0.989. The results showed the significant influence of the vesicle size formulation parameters. The adjusted R^2^ value was 0.976, which is higher than the predicted R^2^ value of 0.769, indicating that the particle size model was less predictive than other responses. This may be attributed to the increased sensitivity of the vesicle size to the composition of the formulation, the distribution of the surfactants, the arrangement of cholesterol, and the preparation parameters. The disparity in particle size distribution can be sufficiently elucidated by the PDI model, yielding an F-value of 95.32 and an R^2^ of 0.9906. The predicted R^2^ was very close to the adjusted R^2^, indicating that the model was very predictive. Low PDI values are important because they indicate consistent vesicle distribution, reduced aggregation, and improved reproducibility of the formulation. The zeta potential model showed high predictive power and significance, with a maximum F-value of 963.20 and an R^2^ of 0.9991. The characteristics of the edge activator are the primary reason for the significant impact. The anionic surfactant sodium cholate demonstrated markedly more negative zeta potential values, while the non-ionic surfactant Tween 80 revealed relatively lower negative values. The moderately negative zeta potential of TPGS was consistent with the steric stabilization of its PEG chains. Optimization was carried out to minimize the particle size and PDI, and to maximize EE% and zeta potential. Selection assumed that an optimal nanovesicular formulation should have a narrow particle-size distribution, high drug loading, small nanosized vesicles, and sufficient stability against aggregation [118]. The optimized formula contained 68 mg of edge activator, 55 mg of cholesterol, and TPGS. The formula indicated a particle size of 198.9 ± 0.98 nm, an EE% of 88.9 ± 0.45%, a PDI of 0.16 ± 0.01, and a zeta potential of −23.1 ± 0.35 mV. The optimized formulation contained TPGS, a multifunctional surfactant, solubilizer, stabilizer and absorption enhancer. The lipophilic vitamin E moiety can interact with the oil phase and NTB, while the hydrophilic PEG chain enhances steric stabilization and dispersion. Cholesterol increased the stability of vesicular bilayers and drug entrapment. However, the concentration of cholesterol should be optimized, as an increase in concentration may lead to an increase in the rigidity and size of the vesicle. The ANOVA results for the quadratic models indicated that they were statistically significant and reliable for optimizing NTB-OOs. The optimized formulation showed a good compromise between an acceptable nano-scale size, high entrapment efficiency, good homogeneity, and good colloidal stability. Therefore, the optimized formulation of NTB-OOs is a promising nanovesicular system for improved delivery of NTB. However, further in vitro studies such as stability, cytotoxicity, cellular uptake, and in vivo release are necessary to confirm its therapeutic potential.

#### 3.2.4. Evaluation of the Optimization of Neratinib-Loaded Ocimum Oil Vesicles (NTB-OOs)

##### Transmission Electron Microscope (TEM)

**Figure 5:**
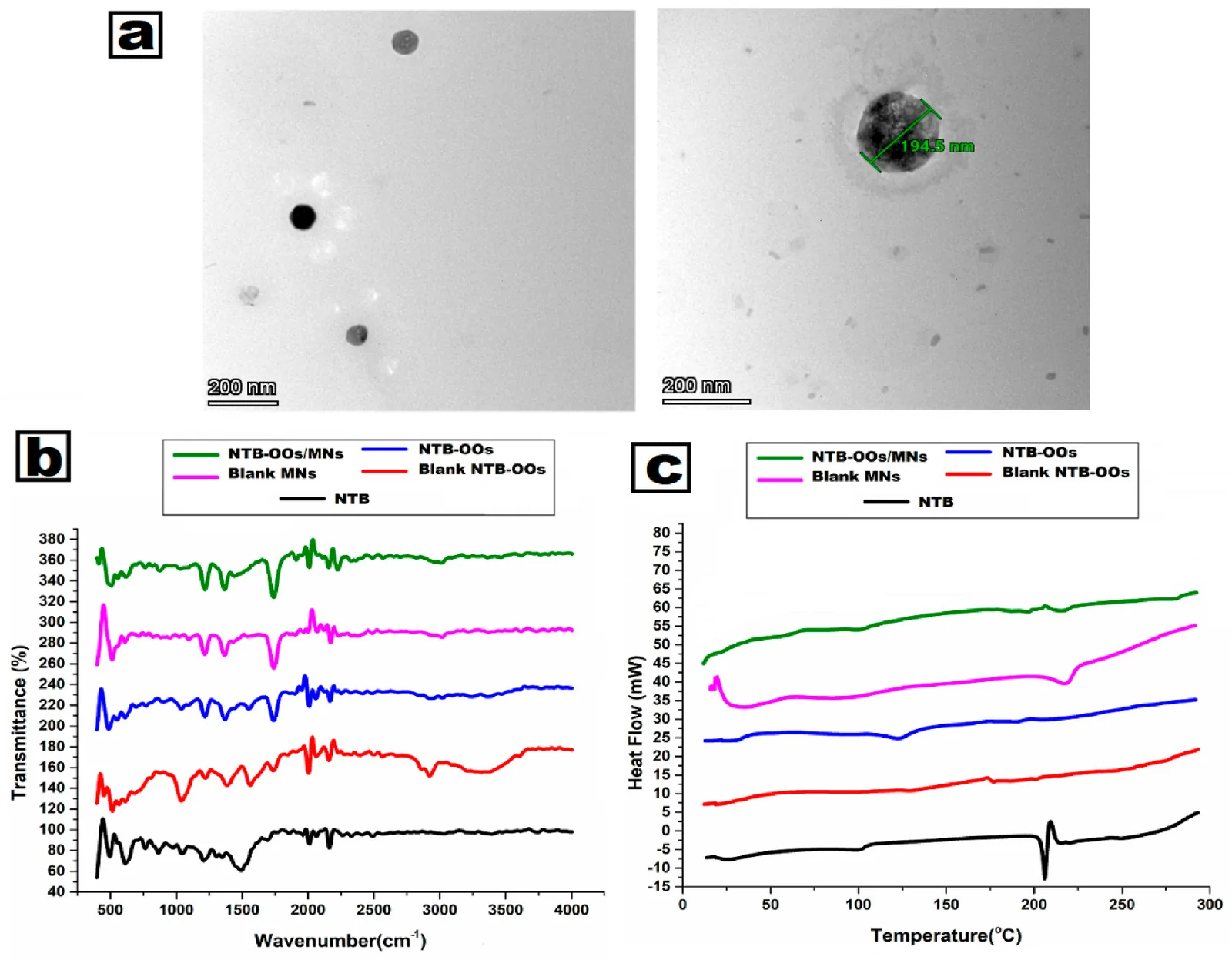
(**a**) TEM micrograph for optimized neratinib-loaded *Ocimum* oil nanovesicles formulations (NTB-OOs), (**b**) Fourier Transform Infrared Spectroscopy, and (**c**) Differential Scanning Calorimetry for NTB, Blank *Ocimum* oil nanovesicles (Blank-OOs), neratinib-loaded *Ocimum* oil nanovesicles formulations (NTB-OOs), Blank PVA/PVP–Propolis Dissolving Microneedles, PVA/PVP–Propolis Dissolving Microneedles loading freeze-dried neratinib-loaded *Ocimum* oil vesicles (NTB-OOs).

The TEM micrograph of optimized NTB-OOs exhibited distinct, nearly spherical nanovesicles with well-defined contours and no visible aggregation, as shown in Figure 5a. The measured vesicle diameter was ~194.5 nm, closely matching the particle size measured by dynamic light scattering for the optimized formulation, 198.9 ± 0.98 nm. This contrast confirms the successful production of nanovesicles. The marginal variation in the sizes measured by TEM and DLS can be attributed to the difference between the diameter measured by TEM in the dry state and the hydrodynamic diameter of the particles in an aqueous suspension as measured by DLS. Transmission electron microscopy (TEM) is commonly used to confirm the morphology, size, and internal structure of nanoparticles in colloidal drug delivery systems [59]. The spherical nanosized morphology is important, as it allows better drug dispersion, improved interaction with biological membranes, and more uniform drug release through small, uniform vesicles [119]. The TEM results indicated that the NTB-OOs nanovesicles were successfully synthesized and stable.

##### Fourier Transform-Infrared Spectroscopy (FTIR)

Figure 5b shows the FTIR spectrum of pure NTB, showing characteristic absorption bands at about 3487, 3244, 3000, 1718, 1481, 1013, and 622 cm^−1^. The bands are mainly attributed to N–H/O–H stretching, aromatic/aliphatic C–H stretching, carbonyl or conjugated C=N/C=O stretching, aromatic skeletal vibrations, C–O/C–N stretching, and fingerprint region vibrations. In a previous study of NTB delivery, FTIR was also used to confirm NTB loading, reporting a characteristic absorption band at ~3427 cm^−1^, further supporting the assignment of the high-wavenumber drug band to N–H/O–H-related stretching. For Blank-OOs and NTB-OOs, the spectra were mainly characterized by bands associated to lipid/oil, such as 2955–2904 cm^−1^ (aliphatic C–Hstretching), 1731 cm^−1^ (ester/carbonyl groups), 1577–1640 cm^−1^ (C=C/aromatic or conjugated vibrations), 1385–1378 cm^−1^ (CH_2_/CH_3_ bending) and 1218–1045 cm^−1^ (C-O/C-O-C stretching) [48,120]. These assignments are in concordance with reported vibrational features of *Ocimum* oils. For example, the bands at about 2834/2967, 1611/1639, 1510–1511, 1436, 1301, 1244, and 1176 cm^−1^ were reported for *Ocimum basilicum* in previous spectroscopic work and assigned to C–H, C=C, aromatic, CH bending, and ether/C–O vibrations. Critically, no additional new bands were observed; the sharp NTB bands were significantly diminished or obscured by the oil/polymer matrix [121]. This implies that NTB was either physically entrapped or molecularly dispersed within the nanovesicles and then incorporated into the oil nanovesicles without any apparent chemical incompatibility.

##### Differential Scanning Calorimetry (DSC)

The DSC curve of Pure NTB is shown in Figure 5c, with a typical thermal event at approximately 202.6 °C, attributed to the melting and decomposition of NTB. A previous study on NTB nanoparticles reported a significant DSC endotherm at 203 °C in the unmodified NTB. Patent information indicates that anhydrous NTB Form exhibits a DSC onset between 196 and 204 °C, during which melting and decomposition occur. No distinct NTB melting peak was observed in NTB-OOs and NTB-OOs/MNs. In NTB-OOs, a broad thermal event was observed around 121.4 °C, and in NTB-OOs/MNs, weak, broad events were observed at around 193.0 and 214.1 °C, without recovering the sharp NTB crystalline peak [20,31]. This implies that NTB was no longer present as a crystalline drug phase after encapsulation and loading into the microneedles but was likely molecularly dispersed or converted to an amorphous or less-crystalline form within the oil vesicles and the polymeric microneedle matrix. Blank MNs showed a broad low-temperature event at ~35.0 °C, which could be associated with residual or bound moisture or polymer relaxation, and a second event at ~216.0 °C, which could be associated with the thermal transition or degradation properties of the PVA/PVP matrix [34]. This is consistent with previous thermal studies showing low-temperature events in PVA due to the loss of water/volatiles, and a high-temperature transition at about 200 °C due to thermochemical changes, softening, or melting with decomposition. The use of propolis may improve the expansion and thermal stability of the polymeric system. The propolis-loaded PVA nanoparticles were reported to be more thermostable than the plain PVA nanoparticles.

### 3.3. Stability Study of the Optimized Neratinib-Loaded Ocimum Oil Vesicles (NTB-OOs)

Table 7 shows the stability study of the optimized NTB-OOs formula. The formula showed good stability for 6 months when stored at ambient and refrigerated temperatures (4 ± 1 °C). The formulation demonstrated a high level of entrapment efficiency after 24 h, with EE values of 88.9 ± 0.45% under refrigerated conditions and 87 ± 0.98% under ambient conditions. EE decreased slightly to 81 ± 0.65% and 80 ± 0.98% after six months, respectively. This slight decrease indicates that there is little leakage of drug from the vesicles during storage. The particle size increased slightly over time [122,123]. At refrigerated temperatures, PS increased from 198.9 ± 0.98 to 210 ± 0.87 nm, whereas at ambient temperatures, this slight rise could be due to small vesicle distension or limited aggregation. However, the vesicles remained in the nanoscale range, confirming satisfactory physical stability. The PDI values were low during the study, rising from 0.16 ± 0.01 to 0.21 ± 0.02 at refrigerated temperatures and from 0.19 ± 0.08 to 0.23 ± 0.04 at ambient temperatures. All PDI values were <0.30, indicating a relatively homogeneous particle-size distribution in the formulation. The ZP values were always negative during the storage period. ZP shifted from −23.1 ± 0.35 mV to −19.1 ± 0.41 mV under refrigerated conditions and from −22 ± 0.98 mV to −19 ± 0.18 mV under ambient conditions after 6 months. A small decrease in surface charge could be attributed to the reorganization of vesicle components during storage, but the residual negative charge contributed to the colloidal stability. The optimized NTB-OOs formulation exhibited acceptable stability for six months. The increase in particle size and zeta potential was slightly lower and better maintained during refrigerated storage, indicating a slight improvement in reliability [124,125]. The results indicate that NTB-OOs can preserve NTB and maintain a uniform vesicular structure and nanoscale dimensions during storage.

### 3.4. Physical Characteristics of PVA/PVP–Propolis-Dissolving Microneedles Loading Freeze-Dried Neratinib-Loaded Ocimum Oil Vesicles (NTB-OOs/P-MNs)

Freeze-dried NTB-loaded *Ocimum* oil vesicles were incorporated in a hydrophilic polymeric matrix of PVA, PVP K90, and propolis to effectively prepare PVA/PVP–propolis dissolving microneedles. Three formulations were prepared with different polymer/propolis ratios. Propolis-MNs1 consisted of 20% PVA, 25% PVP K90, and 0.5% propolis. Propolis-MNs2 consisted of 25% PVA, 20% PVP K90, and 1% propolis. Propolis-MNs3 consisted of 30% PVA, 15% PVP K90, and 1.5% propolis. A PDMS mold containing 100 conical cavities arranged in a 10 × 10 grid was used to fabricate the microneedles. PDMS microneedles were obtained by vacuum-assisted filling followed by a backing layer, overnight drying, and storage in desiccators [126,127].

#### 3.4.1. Mechanical Strength and Penetration Capability Test

**Figure 6:**
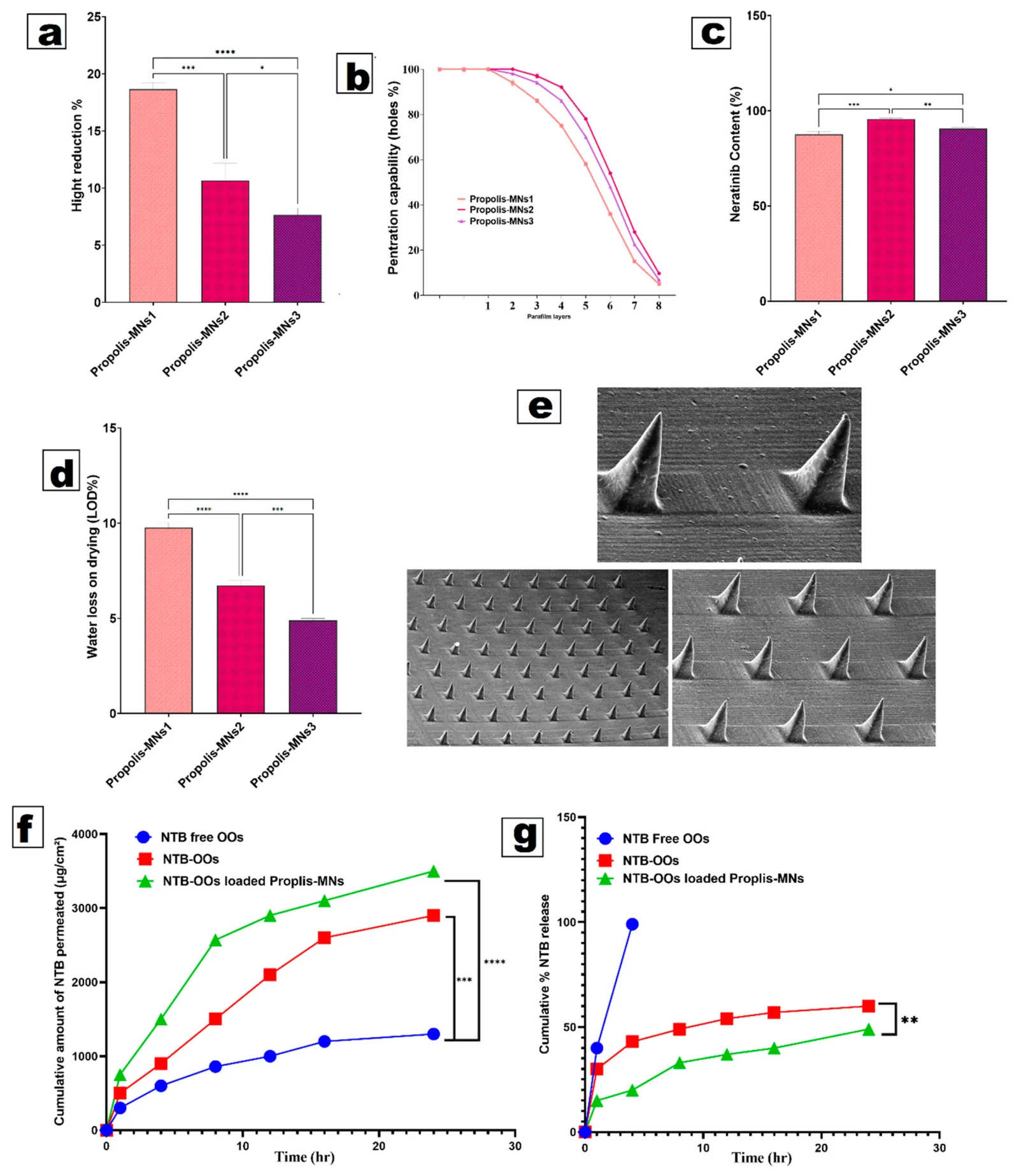
(**a**–**d**) Physicochemical Characterization of PVA/PVP–Propolis Dissolving Microneedles loading freeze-dried neratinib-loaded *Ocimum* oil vesicles (NTB-OOs). (**a**) Mechanical integrity assessment, (**b**) skin Penetration Capability, (**c**) drug content, (**d**) water loss on drying (LOD). (**e**) Scanning electron microscopy, (**f**) in vitro NTB release, (**g**) skin ex vivo permeation study for NTB suspension, NTB-OOs, and NTB-OOs/PVA-PVP Propolis-MNs. Data are presented as mean ± SD (n = 3). Statistical significance was analyzed using one-way ANOVA followed by post hoc comparisons; * *p* < 0.05, ** *p* < 0.01, *** *p* < 0.001, and **** *p* < 0.0001.

Figure 6a shows that the mechanical strength was evaluated by measuring the % reduction in microneedle height after compression. The lower the height reduction, the better the resistance to deformation and the better the retention of the sharp needle shape when inserted. The largest reduction in height was observed in Propolis-MNs1, at around 18–19%, suggesting the lowest mechanical integrity. Propolis-MNs2 exhibited deformation of around 10–11%, while Propolis-MNs3 showed a smaller reduction in height of around 7–8%, thereby confirming its better mechanical characteristics [128,129]. This behavior can be explained by the formulation’s composition. Probably, the increase in PVA concentration from 20% to 30% resulted in a more robust, denser polymeric network, thereby increasing the rigidity of the needle. PVA is widely used in microneedle dissolution because of its good film-forming properties and mechanical strength. The increased amount of PVP K90 used in Propolis-MNs1 may have increased hydrophilicity and water uptake, resulting in a softer, more plastic matrix. In previous studies, it was reported that PVA/PVP-based microneedles have sufficient mechanical strength for insertion and that the polymer ratio is important for mechanical and dissolution properties. The matrix reinforcement may also be improved with increased propolis concentration. Propolis is known to contain phenolic and flavonoid compounds that can interact with polymer chains via hydrogen bonding, leading to a denser structure. A previous study on Brazilian green propolis polymeric microneedles developed from PVA/PVP based blends showed that the propolis-containing microneedles could be successfully fabricated and mechanically tested, confirming the potential of propolis as an active compound for polymeric microneedle systems.

Figure 5b shows that the ability to penetrate skin was tested using the Parafilm^®^ M model. The method used involved placing eight layers of Parafilm^®^ on top of each other to reach a thickness of approximately 1 mm and pressing the microneedles with a standardized force of 32 N for 30 s. This simulated manual thumb pressure during application. All formulations penetrated well into the upper layers of Parafilm, but penetration became progressively less in the deeper layers. Propolis-MNs3 showed the highest penetration efficiency, followed by Propolis-MNs2 and Propolis-MNs1. This result is in agreement with the data on mechanical strength, since microneedles with lower deformation are more effective at keeping sharp tips and penetrating deeper layers. Parafilm^®^ M has been proposed and validated as an effective artificial membrane model for the initial microneedle insertion experiments [32]. As its folded structure can mimic the thickness of the skin, the depth of insertion can be determined by counting the number of perforations in each layer. The present results showed that Propolis-MNs3 had sufficient mechanical strength to overcome insertion resistance and form microchannels for the deposition of NTB-OOs into the skin.

#### 3.4.2. Determination of Drug Content

Figure 5c shows the drug content analysis, confirming the successful incorporation of NTB-OOs into the microneedle matrix. Propolis-MNs1 showed about 88–89% drug content, Propolis-MNs2 showed the highest, at about 95–96%, and Propolis-MNs3 showed about 90–91%. The higher drug concentration in Propolis-MNs2 could be associated with the intermediate PVA/PVP ratio, which may have balanced viscosity and polymer flexibility, thereby promoting the uniform dispersion of freeze-dried NTB-OOs in the microneedle-tip solution. Propolis-MNs3 exhibited significant drug content, enhanced mechanical strength, reduced height reduction, reduced loss on drying (LOD), and enhanced penetration [71].

#### 3.4.3. Dissolution Time Assessment

The evaluation of dissolution time is significant, as microneedles must maintain mechanical integrity before insertion and dissolve rapidly after insertion to release their payload. Microneedles were inserted into excised mouse skin, and a 5 g flat weight was applied to mimic long-term adhesion. The patches were removed at 2, 4, 6, and 8 min and examined under light microscopy to observe dissolution of the needle tips. The dissolution-time design indicates that the reduction of needle length with increasing time is due to the hydration and dissolution of the PVA/PVP–propolis matrix after contact with the fluid of the skin. Because of the higher concentration of PVP K90 and the lower PVA/propolis ratio, Propolis-MNs1 is expected to dissolve faster. PVP is highly water-soluble and has been reported to facilitate rapid microneedle dissolution. Previous research has demonstrated that PVP-based dissolving microneedles can dissolve within 10 to 180 s, depending on the polymer’s molecular weight and formulation, whereas another study has shown that rapidly dissolving polymeric microneedles can dissolve within the skin in approximately one minute. In contrast, Propolis-MNs3 with the highest concentrations of PVA and propolis is expected to have a slower dissolution rate and to show improved structural integrity. This balance is good, as too fast dissolution prior to adequate insertion can lessen delivery efficacy, whereas too slow dissolution may necessitate extended patch application. A previous study showed that higher PVA concentration improved the mechanical strength of microneedles but inhibited dissolution, consistent with the trend observed with higher-PVA formulations. Therefore, if the dissolution-time table shows that Propolis-MNs3 dissolves gradually and reaches near-complete or sufficient tip dissolution by 8 min, it would support its designation as the optimized formulation. Among them, Propolis-MNs3 is the best choice for balancing mechanical strength before insertion and dissolution after insertion [34,130]. The dissolution behavior explains the controlled in vitro release profile of NTB-OOs-loaded propolis microneedles, in which the drug was released at a slower rate than from free NTB and NTB-OOs, due to the additional diffusion barrier formed by the dissolving polymeric matrix.

#### 3.4.4. Water Loss During Drying (LOD) Test

Figure 6d shows the water loss during drying; the decrease in water loss during drying was proportional to the increase in PVA and propolis concentrations. Propolis-MNs1 had the highest limit of detection (LOD) (around 9–10%), followed by Propolis-MNs2 (around 6–7%). The lowest value was found with Propolis-MNs3 (around 5%). This shows that Propolis-MNs3 showed the lowest residual moisture after drying. Excess water should be minimized to prevent plasticizing the polymer matrix, softening the microneedles, and reducing insertion efficacy. The reduced height reduction and increased penetration efficacy of Propolis-MNs3 are explained by the reduced LOD. The improved drying features can be attributed to the higher concentration of PVA/propolis, resulting in a denser, more rigid matrix [131,132]. Earlier studies of polymeric microneedles containing propolis showed that increasing the concentration of propolis extract affected mechanical properties and improved the hardness of the microneedle.

The PVA/PVP–propolis microneedles were prepared using three different polymer/propolis formulations. The lowest concentration of PVA and the highest concentration of PVP were found in propolis-MNs1 (20% PVA, 25% PVP, and 0.5% propolis). Propolis-MNs2 was an intermediate formulation, with 25% PVA, 20% PVP, and 1% propolis, and Propolis-MNs3 was the formulation with the highest concentrations of PVA and propolis and the lowest concentration of PVP, 30% PVA, 15% PVP, and 1.5% propolis. The gradual increase in PVA and propolis, with a simultaneous decrease in PVP, clearly influenced the mechanical strength, drying properties, penetration efficacy, and delivery performance of the microneedles [30].

#### 3.4.5. Characterization of Optimized PVA/PVP–Propolis Dissolving Microneedles Loading Freeze-Dried Neratinib-Loaded Ocimum Oil Vesicles (NTB-OOs/P-MNs)

##### Scanning Electron Microscopy (SEM)

Figure 6e shows SEM analysis confirming the successful fabrication of the optimized NTB-OOs/P-MNs. The microneedles were uniform, conical, and regularly arranged, with sharp tips, intact bases, and no visible cracks, fractures, or collapse. The morphological characteristics are important for successful microneedle insertion, as blunt or broken microneedles may not penetrate the stratum corneum. The SEM results corroborate the mechanical and penetration results. The acute conical geometry implies that freeze-dried NTB-OOs were incorporated into the polymeric matrix without affecting the microneedle formation. Studies of previous microneedles have suggested that maintaining intact geometry, sharp tips, and sufficient mechanical integrity are critical for efficient insertion and reproducible drug delivery [31,34].

##### Fourier Transform Infrared (FTIR) Spectroscopy

Figure 5b shows the FTIR spectra of Blank PVA/PVP–propolis microneedles and NTB-OOs-loaded microneedles, and blank microneedles loaded with NTB-OOs. The FTIR spectrum of the blank PVA/PVP–propolis microneedles exhibited the characteristic bands of the polymeric-propolis matrix. The principal observed peaks were located at approximately 3350–3450 cm^−1^, 2920–2980 cm^−1^, 1730–1740 cm^−1^, 1640–1660 cm^−1^, 1370–1450 cm^−1^, 1210–1240 cm^−1^, 1040–1080 cm^−1^, 880–900 cm^−1^ and 520–550 cm^−1^. The large band between 3350 and 3450 cm^−1^ is attributed to the O–H stretching of polyvinyl alcohol (PVA) and the phenolic hydroxyl groups of propolis. The band at 2920–2980 cm^−1^ is assigned to aliphatic C–H stretching. The peak at 1730–1740 cm^−1^ may originate from residual carbonyl or ester groups from the PVA or propolis ingredients, while the band at 1640–1660 cm^−1^ is mainly attributed to the C=O stretching of the pyrrolidone ring in PVP. Previous studies on PVA/PVP hydrogels have shown that the O–H stretching of PVA occurs at about 3330 cm^−1^, the C–H stretching is at about 2920 cm^−1^, carbonyl-related bands of PVA are in the range 1713–1732 cm^−1^, and the C=O stretching of PVP is at about 1655 cm^−1^, which support these assignments. The peaks at 1370 to 1450 cm^−1^ can be attributed to CH bending vibrations; the bands between 1210–1240 cm^−1^ and 1040–1080 cm^−1^ can be attributed to C-O, C–O–C and C-N stretching vibrations of PVA, PVP and propolis [2,33]. The spectrum of unoccupied MNs confirms the successful formation of a composite PVA/PVP–propolis matrix. Broad and overlapping bands are indicative of the presence of hydrogen bonding between hydroxyl groups of PVA, carbonyl groups of PVP and phenolic compounds of propolis. Previous studies on propolis-loaded polymeric microneedles indicated that matrices based on PVA/PVP are suitable delivery platforms for propolis and that propolis loading can affect the mechanical properties of the microneedles. The FTIR spectrum of NTB-OOs-loaded PVA/PVP–propolis microneedles showed the main characteristic bands of the unmodified microneedle matrix with slight changes in intensity and position. The main bands were found in the ranges of 3350–3450 cm^−1^, 2920–2975 cm^−1^, 1730–1740 cm^−1^, 1600–1650 cm^−1^, 1370–1450 cm^−1^, 1210–1220 cm^−1^, 1045–1060 cm^−1^, 885–895 cm^−1^ and 526–540 cm^−1^. The broad O–H band continuity in the range 3350–3450 cm^−1^ also indicated the persistence of the hydrogen-bonding network of PVA/PVP/propolis after the incorporation of freeze-dried NTB-OOs. The bands at 2920–2975 cm^−1^ can be attributed to the C-H stretching from the polymeric matrix and lipid or oil components of the nanovesicles. The peak around 1730–1740 cm^−1^ is probably due to the carbonyl or ester groups present in the polymers, which are constituents of propolis and components of vesicular oil. The bands at 1210–1220 cm^−1^ and 1045–1060 cm^−1^ are assigned to C-O/C-N/C-O-C stretching vibrations. After incorporating NTB-OOs, no new intense peaks were observed compared with the unfilled microneedles. Thus, the nanovesicles were incorporated into the microneedle matrix primarily by physical entrapment, not by any chemical reaction. The characteristic peaks of NTB were not clearly observed in the microneedle spectrum, probably because NTB was diluted in the polymeric matrix, overlapped with the peaks of PVA/PVP/propolis, and encapsulated in the oil vesicles [72]. This result confirms the compatibility of NTB-OOs with the PVA/PVP–propolis matrix. In previously studied PVA/PVP composite systems, the broadening and shifting of the FTIR peaks were attributed to interactions between polymer chains, particularly hydrogen bonding between the hydroxyl groups of PVA and the carbonyl groups of PVP. Similar matrix-dominated bands were observed in current-loaded microneedles, suggesting that NTB-OOs did not disrupt the polymeric network. This supports the interpretation that propolis was not only physically dispersed but also may interact with polymer–propolis interactions within the matrix.

##### Differential Scanning Calorimetry (DSC)

The DSC thermogram of the blank PVA/PVP–propolis microneedles showed broad thermal events, not distinct crystalline peaks. A typical low-temperature effect was observed at about 35–45 °C due to the evaporation of adsorbed or bound moisture and the relaxation of the hydrophilic polymeric matrix. This is expected because of the water retention ability of both PVA and PVP through hydrogen bonding. A separate thermal transition appeared at approximately 210–220 °C. This event could be related to changes in the polymeric matrix, the melting or softening of PVA-rich domains, the onset of thermal degradation, or the rearrangement of the PVA/PVP–propolis network. Earlier studies on PVA/PVP hydrogels have demonstrated that PVP exhibits a broad thermal event at approximately 98 °C, which is associated with the evaporation of residual solvent or low-molecular-weight components. In contrast, pure PVA exhibited a melting point at around 191 °C [33,34]. In PVA/PVP composites, these transitions might shift or broaden due to polymer–polymer interactions and miscibility within the matrix. Thus, the unfilled microneedle DSC profile indicates the formation of a predominant amorphous or semi-amorphous polymer matrix. The lack of distinct thermal peaks indicates that the blank PVA/PVP–propolis microneedles were not in the majority crystalline materials. This is advantageous for microneedle dissolution since an amorphous hydrophilic matrix can hydrate and dissolve upon insertion into the DSC of NTB-OOs-loaded MNs. The DSC thermogram of NTB-OOs-loaded microneedles of PVA/PVP–propolis showed broad thermal events, instead of sharp melting peak of the drug. The main estimated events occurred at about 45–60 °C, 190–195 °C, and 210–215 °C. The low-temperature event in the range 45–60 °C is attributed to the evaporation of moisture and relaxation of the hydrated polymeric structure. The events at 190–195 °C and 210–215 °C can be related to the transition of the PVA/PVP matrix and the thermal reconfiguration of the polymer–propolis–vesicle system. The large size of these events suggests that the loaded microneedles were primarily amorphous or molecularly dispersed. The thermogram of NTB-OOs-loaded microneedles did not show the characteristic crystalline melting/decomposition peak of pure NTB. This means that NTB was not present as a separate crystalline phase after incorporation into the nanovesicles and microneedle matrix. NTB was likely molecularly dispersed, amorphized, or highly entrapped within the vesicles of *Ocimum* oil and then incorporated into the PVA/PVP–propolis matrix. This interpretation is consistent with previous studies on polymeric hydrogels where the lack of a distinct crystalline peak was used as evidence that a component was molecularly dispersed and did not recrystallize within the polymeric matrix. This is consistent with results on PVA/PVP composite systems, where interactions between polymers can broaden and shift DSC transitions, rather than leading to separate sharp transitions of each component. DSC results showed that incorporating NTB-OOs into the microneedles did not lead to drug crystallization or any visible incompatibility. The thermogram reveals the successful incorporation of NTB-OOs into a compatible polymeric matrix. The importance is that the drugs in amorphous or molecularly dispersed state in the nanovesicle loaded microneedle system may improve the release and permeation properties.

### 3.5. Drug Release Studies of Polydispersity Ind

#### 3.5.1. In Vitro Drug Release Study and Kinetic Models

Figure 6f The in vitro release profiles showed distinct patterns among free NTB, NTB-OOs, and NTB-OOs-loaded propolis microneedles. The free NTB was released fast and almost completely during the first hours. NTB-OOs exhibited a more sustained release profile, with ~60% released after 24 h. Propolis microneedles loaded with NTB-OOs showed the slowest release, reaching about 48–50% after 24 h. The prolonged release is attributed to the synergistic regulation of the vesicular system and the polymeric microneedle matrix. NTB has to be diffused first from the *Ocimum* oil vesicles and then through the hydrated PVA/PVP–propolis matrix. Thus, microneedle formulation can act as both a skin-penetrating device and a controlled-release system. This is in line with previous findings that dissolving microneedles can be engineered to provide either a rapid or sustained release depending on the polymer composition, hydration, and matrix architecture.

The kinetic analysis confirmed this behavior, since the NTB suspension presented the highest R^2^ value (0.978) and the best fit to the first-order model. The first-order release rate depends on the remaining drug concentration, so the release rate is initially fast and then slows as the amount of drug available decreases. Conversely, NTB-OOs showed a more consistent release profile, with an estimated 60% release at 24 h. The slower release of NTB-OOs compared to free NTB confirms the successful entrapment of NTB in the *Ocimum* oil vesicular system. Here, the release of NTB was controlled by diffusion from the internal oil/vesicular phase to the external aqueous medium. The vesicular membrane and the oily domain served as a diffusion barrier, resulting in a sustained release profile and reduced burst release. Such behavior is desirable, as it has the potential to extend the availability of NTB and reduce rapid drug loss from the delivery site. The kinetic data also support this explanation. The NTB-OOs were best fitted by the Korsmeyer–Peppas model (R^2^ = 0.989), and the Higuchi model was also close (R^2^ = 0.987), as shown in Table 8. This implies that diffusion was an important mechanism but not the only controlling factor. The Peppas exponent n = 0.524 indicates anomalous or non-Fickian transport. Thus, the release of NTB from OOs was mediated by multiple mechanisms like drug diffusion, vesicular rearrangement, and partitioning of NTB from the oil vesicular phase into the surrounding medium. The NTB-OOs loaded with PVA/PVP–propolis microneedles exhibited the slowest and most controlled release, with approximately 48–50% released after 24 h. This delayed release is due to the formulation’s double-barrier structure. At first, NTB was enclosed in vesicles of *Ocimum* oil. Secondly, these vesicles were encapsulated in the PVA/PVP–propolis microneedle matrix. Post-hydration, the polymeric matrix swells and slowly dissolves, resulting in the gradual release of NTB-OOs, followed by diffusion of NTB from the vesicles. Thus, the NTB release from the microneedle formulation was controlled by polymer dissolution/relaxation and vesicle-mediated diffusion. Kinetic analysis indicated that propolis microneedles loaded with NTB-OOs best fitted the zero-order model with the highest R^2^ value of 0.986. Zero-order release is highly beneficial for transdermal and local drug delivery methods as it avoids rapid burst release and offers controlled drug input, as it signifies an approximately constant drug release rate over time. The Peppas exponent value of n = 0.892 indicated that polymer relaxation, swelling, and dissolution also significantly affected the release mechanism. Thus, the PVA/PVP–propolis microneedle matrix was not only a carrier for skin insertion but also a controlled release platform. The results were consistent with previous studies on NTB formulation. Aleanizy et al. prepared neratinib-loaded PAMAM dendrimer nanocapsules and observed a similar in vitro release pattern from both plain and trastuzumab-conjugated dendrimers [133]. The release rate was slightly lower for trastuzumab-conjugated dendrimers, possibly due to drug loss or a stronger association with the carrier during conjugation. Similarly, Lila et al. prepared NTB-loaded biotin-chitosan-decorated multiwalled carbon nanotubes and found that NTB release was pH-dependent, with greater release at acidic pH [134]. Thus, the release behavior of NTB is greatly influenced by the nanocarrier structure and the surrounding medium. Other delivery systems for NTB also offers controlled release. Rahamathulla et al. prepared effervescent floating matrix tablets of NTB and reported that the optimized formulation exhibited 100% release within 12 h, with a sustained-release profile and a non-Fickian mechanism, demonstrating that polymeric matrices can effectively control NTB release [135]. Furthermore, NTB maleate-loaded lipid nanoparticles exhibited an initial burst release followed by sustained release for up to 12 h. This finding supports the utility of lipid-based nanocarriers for sustained release of NTB. The prolonged in vitro release of genipin-crosslinked albumin nanoparticles containing NTB and silibinin further confirms that nanoencapsulation is a practical approach to enhance NTB delivery performance and prolong its release. The in vitro release results demonstrated the rapid release of free NTB, the sustained vesicle-controlled release of NTB-OOs, and the most controlled release profile of NTB-OOs-loaded PVA/PVP–propolis microneedles. The kinetic study showed that the optimized microneedle formulation obeyed the best-fit zero-order model, indicating a controlled, nearly constant release of NTB. This validates the efficacy of combined dissolving propolis microneedles and NTB-OOs in converting NTB release from rapid, concentration-dependent to sustained, controlled delivery.

#### 3.5.2. Ex Vivo Permeation Study

Figure 6g shows the ex vivo skin permeation performed using vertical Franz diffusion cells to compare the permeation of NTB from NTB suspension, NTB-OOs, and NTB-OOs-loaded PVA/PVP–propolis microneedles. Excised rat skin was mounted between the donor and receptor compartments, with the stratum corneum facing the donor side. The receptor medium consisted of a phosphate buffer (pH 7.4) containing 0.5% Tween 80, and was maintained at 32 ± 0.5 °C to mimic the skin surface temperature. Samples were withdrawn at pre-determined time intervals and analyzed by HPLC. The NTB suspension exhibited the lowest cumulative permeation, approximately 1300 µg/cm^2^, after 24 h. This limited permeation is attributed to the stratum corneum’s strong barrier function and the limited ability of free NTB molecules to penetrate the dense, lipid-rich skin layer. For this reason, the suspension did not deliver a large amount of NTB transdermally. NTB-OOs significantly increased the NTB permeation to approximately 2900 µg/cm^2^ after 24 h, which was about 2.2-fold higher than that of the NTB suspension. This enhancement might be attributed to the nanoscale vesicular structure of the vesicles of *Ocimum* oil. Nanovesicles can improve drug-skin interaction, enhance drug solubilization, and improve drug partition into dermal layers. Lipidic vesicular components can interact with the lipid matrix of the stratum corneum and thus improve the diffusion of the drug through the skin. Earlier studies showed that nanovesicular systems increase skin penetration by altering or fluidizing the lipid structure of the stratum corneum and enhancing drug deposition in the skin. The NTB permeation was the highest for NTB-OOs-loaded PVA/PVP–propolis microneedles. The permeated NTB was almost 3500 µg/cm^2^ after 24 h, indicating 2.7-fold and 1.2-fold increases compared with NTB suspension and NTB-OOs, respectively. This result confirms that the microneedle system conferred an additional permeation-enhancing effect compared with nanovesicles alone. The microneedles pierced the stratum corneum by creating microchannels in the skin. The dissolvable PVA/PVP–propolis matrix hydrated and disintegrated upon insertion, releasing NTB-OOs directly into or adjacent to these microchannels. The higher concentration of NTB in the receptor compartment shows enhanced ex vivo transdermal penetration of the microneedle formulation. This finding corroborates the disruption of the stratum corneum by the microneedles and the subsequent vesicle-mediated transport. In the present Franz-cell study, however, the NTB retained in specific strata of skin was not measured and therefore intradermal drug distribution or localized tumor concentration cannot be conclusively determined. This mechanism is consistent with prior microneedle work showing that microneedle insertion disrupts the stratum corneum and generates aqueous microchannels that promote the passage of drugs and nanoparticles through the skin. Microneedle-mediated nanoparticle delivery is considered a promising approach, combining the physical penetration capability of microneedles with the solubilizing and controlled-release properties of nanocarriers [136,137]. These findings are consistent with a prior investigation of NTB that showed neratinib-loaded solid lipid nanoparticles embedded within dissolvable microneedles improved ex vivo penetration into porcine skin, with a cumulative drug release/penetration of 80.71 ± 1.43% over 24 h. In addition, solid-lipid-nanoparticle-embedded dissolving microneedles have shown good mechanical and insertion properties and improved drug retention in the dermal layers, supporting the use of nanocarrier-loaded microneedles for improved transdermal delivery. Ex vivo results showed that PVA/PVP–propolis microneedles loaded with NTB-OOs were more effective than both NTB suspension and NTB-OOs. The increased permeation was mainly attributed to the synergistic effects of microneedle-mediated stratum corneum bypass, vesicle-mediated NTB diffusion, and controlled release from the dissolving polymeric matrix. Hence, NTB-OOs/P-MNs were identified as the best transdermal delivery system, and the zero-order model was the most suitable kinetic model to describe the release of NTB from the optimized microneedle formulation.

### 3.6. In Vitro Cytotoxicity Against MCF-7 Cells

**Figure 7:**
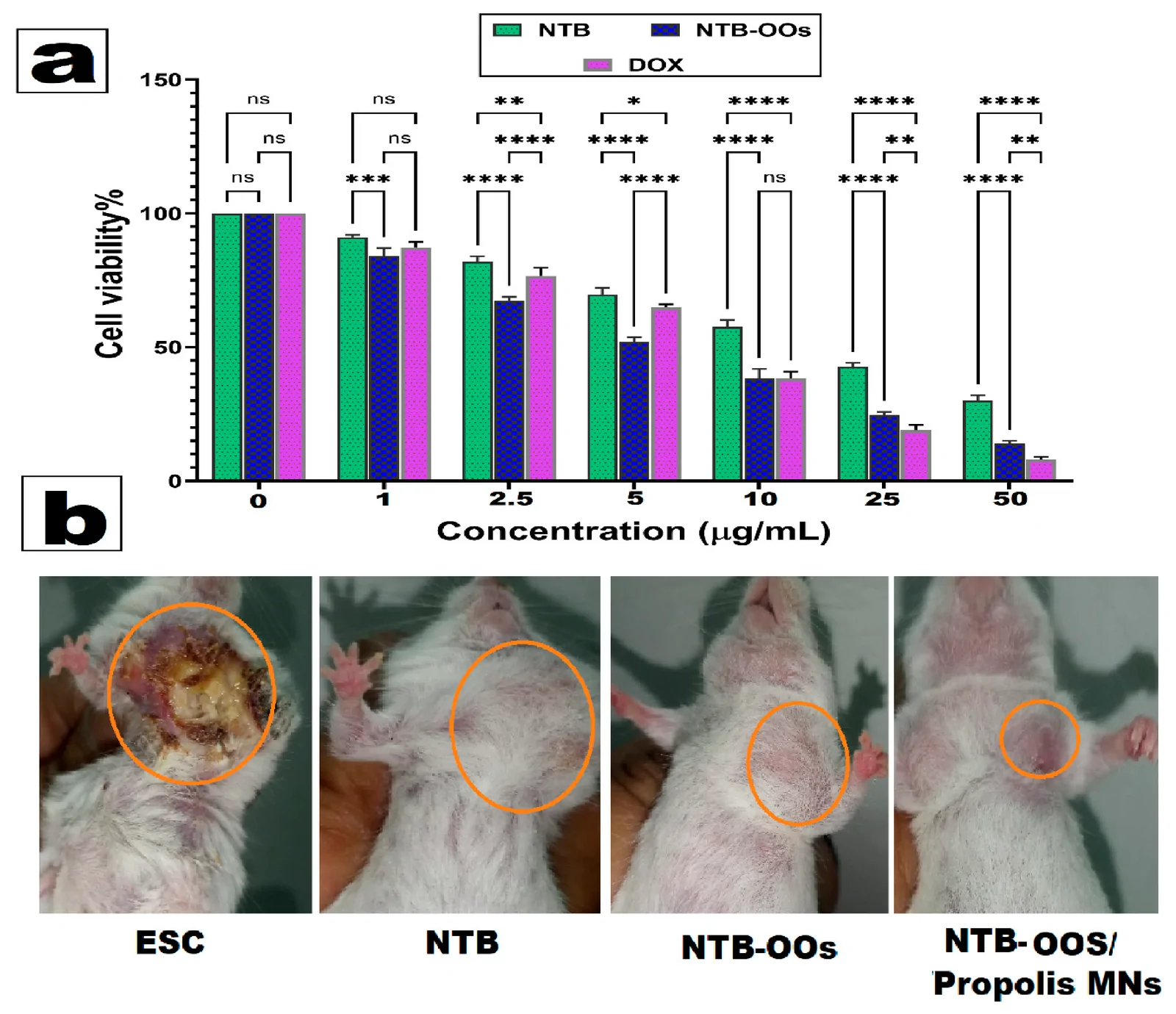
(**a**) In vitro cytotoxicity assessment of neratinib formulations using the human breast adenocarcinoma cell line MCF-7. (**b**) In vivo antitumor efficacy of different neratinib formulations in Ehrlich solid carcinoma-bearing mice, showing representative photographs of excised tumors from each experimental group. Note: * *p* < 0.05, ** *p* < 0.01, *** *p* < 0.001, **** *p* < 0.0001 and ns (not significant).

An in vitro cytotoxicity study was conducted to assess the enhanced anticancer activity of NTB, using *Ocimum* oil-based nanovesicles, against MCF-7 breast cancer cells. NTB is a selective irreversible pan-HER tyrosine kinase inhibitor that targets HER-family receptors, specifically EGFR/HER1, HER2, and HER4. NTB is an irreversible inhibitor of HER receptor autophosphorylation, blocking downstream proliferative and survival pathways and leading to the inhibition of cancer cell growth and induction of cell death. Preclinical studies have shown that NTB is a potent, long-acting inhibitor of HER family signaling that reduces the proliferation of EGFR and HER2-dependent neoplastic cells [138,139]. The present study demonstrated that the free NTB reduced the viability of MCF-7 cells in a concentration-dependent manner, thus confirming its intrinsic antiproliferative effect. Cell viability was decreased from 91.0 ± 1.0% at 1 µg/mL to 30.0 ± 2.0% at 50 µg/mL. This may be due to the inhibition of HER-mediated signaling cascades that control breast cancer cell proliferation. The free NTB showed a moderate cytotoxic effect compared to the nanoformulated drug, due to its low aqueous solubility and inadequate interaction with or uptake by cancer cells. Incorporating NTB into nanovesicles of *Ocimum* oil led to a significant increase in cytotoxicity. NTB-OOs decreased the viability of MCF-7 cells from 84.0 ± 3.0% at 1 µg/mL to 14.0 ± 1.0% at 50 µg/mL. The inhibition effect of NTB-OOs was better than that of free NTB at each concentration. The calculated IC_50_ value decreased from about 11.1 μg/mL for free NTB to about 5.1 μg/mL for NTB-OOs, indicating a 2.2-fold increase in cytotoxic efficiency, as shown in Figure 7a. The lower IC_50_ of NTB-OOs compared to free NTB showed the higher cytotoxic activity of the complete NTB-loaded vesicular formulation. However, the present cytotoxicity experiment did not include vesicles of drug-free *Ocimum oil*, so the relative contributions of enhanced NTB delivery and intrinsic biological activity of the oil-containing carrier cannot be distinguished. The increased effect of NTB-OOs is explained by several formulation-related mechanisms. The oil-vesicular system could increase the NTB solubilization and dispersion, thus increasing the amount of drug available for interaction with cancer cells. Secondly, the nanoscale vesicles may enhance surface interactions with the cell membrane and facilitate cellular uptake. Third, the lipidic structure of the vesicles might interact with the cell membrane, thereby facilitating NTB partitioning into cells. In a previous study of NTB, NTB-loaded solid lipid nanoparticles were reported to exhibit higher cytotoxicity against MCF-7 cells than free neratinib, as evidenced by lower IC_50_ values, indicating that lipid-based nanocarriers enhance NTB cellular delivery and anticancer efficacy [140]. There may have been additional anti-cancer effects from *Ocimum* oil. The cytotoxicity, induction of apoptosis, cell-cycle arrest, and inhibition of tumor growth of *Ocimum* species in various in vitro and in vivo cancer models have been associated with bioactive compounds such as phenolics and terpenoids. A review on the anticancer activity of *basil* (*Ocimum*) reported that basil bioactive compounds can reduce the viability of cancer cells and modulate cell death pathways. Moreover, *Ocimum basilicum* essential oil has been reported to exhibit cytotoxic activity against MCF-7 cells, with an IC_50_ of 80.35 ± 1.17 µg/mL. The enhanced cytotoxicity observed with NTB-OOs could be attributed to the synergistic effects of nanovesicular NTB delivery and the biological properties of the carrier containing *Ocimum* oil. However, it is not possible to quantify the individual contributions of each component from the present experimental design. The sensitivity of MCF-7 cells to anticancer treatment was confirmed by using DOX as a positive control. The strong cytotoxic effects were confirmed by a cell viability of 8.0 ± 1.0% at a DOX concentration of 50 µg/mL. The present study does not mainly compare NTB with DOX because DOX is a classical cytotoxic chemotherapeutic drug, whereas NTB is a targeted tyrosine kinase inhibitor [141]. The major finding is that the NTB-OOs significantly increased the cytotoxic efficacy of NTB, as indicated by decreased cell viability and lower IC_50_ values compared with free NTB. From a formulation perspective, the results validate the efficiency of the synthesized *Ocimum* oil nanovesicles in transforming NTB from a moderately active free drug into a more potent nanoformulated anticancer system. The increase in cytotoxicity at lower concentrations is significant because it may improve local therapeutic efficacy and decrease the dosage required to achieve an anti-cancer effect. However, inclusion of an NTB-OOs group is recommended in the final study to ensure that the main cytotoxic effect is due to NTB loading and not the carrier alone.

### 3.7. In Vivo Study

#### 3.7.1. In Vivo Antitumor Efficacy in Solid Ehrlich Carcinoma Model

An in vivo solid Ehrlich carcinoma study confirmed the in vitro cytotoxicity results. The model was established by the subcutaneous inoculation of Ehrlich carcinoma cells, and treatment was initiated after tumor formation. The experimental groups were treated transdermally with gels of NTB, NTB-OOs, or NTB-OOs/P-MNs on every alternate day for two weeks. The untreated ESC group exhibited the highest intensity of tumor growth, supporting the aggressive progression of the model in the absence of treatment. Treatment with NTB gel reduced tumor burden compared with the untreated group due to the antitumor effects of NTB. NTB gel showed limited efficacy, probably due to insufficient skin penetration of free NTB, which might not reach therapeutic levels in the tumor tissue after topical administration. The NTB-OOs gel exhibited greater antitumor efficacy than the free NTB gel. The enhanced effect of the *Ocimum* oil nanovesicles was attributed to the ability to increase the solubilization of NTB, which protected the drug and enhanced its delivery to the skin and tumor-surrounding tissues. Thus, the nanovesicles likely increased the local concentration of NTB in the tumor region, leading to stronger HER signaling inhibition and greater tumor growth suppression. This interpretation is consistent with the in vitro results, in which NTB-OOs were more effective than free NTB at reducing MCF-7 cell viability. The greatest antitumor activity was demonstrated by NTB-OOs/P-MNs. This formulation combines three therapeutic benefits: the pharmacological activity of NTB, the penetration-enhancing activity of *Ocimum* oil nanovesicles, and the physical penetration ability of dissolving microneedles. Microneedles penetrate the stratum corneum, creating microchannels, which are then dissolved, releasing NTB-OOs in or near skin layers. This allows a more efficient local delivery of the nanovesicles as compared to passive diffusion from the gel. Microneedle-based systems have been widely investigated as minimally invasive platforms that improve local anticancer delivery by increasing drug penetration and drug availability at the target site. Thus, the total antitumor efficacy can be explained by the following sequence: NTB-OOs/P-MNs > NTB-OOs gel > NTB [1,92] gel > untreated ESC group, as shown in Figure 7b. This order demonstrates that the therapeutic response was not only dependent on the anticancer effects of NTB but also greatly impacted by the delivery system. Free NTB showed pharmacological activity, NTB-OOs increased drug availability and cellular/skin interaction, and NTB-OOs/P-MNs optimized local delivery by overcoming the skin barrier and prolonging drug release. Therefore, the optimized formulation improved the biological efficacy and delivery efficiency of NTB. The results indicate that incorporating NTB into Ocimum oil nanovesicles and their entrapment in PVA/PVP–propolis dissolving microneedles represent a promising approach for localized breast cancer therapy. The formulation increased NTB cytotoxicity in MCF-7 cells and improved in vivo antitumor efficacy in the ESC model, likely due to increased solubilization, vesicle-mediated delivery, microneedle-assisted penetration, and sustained local drug release.

#### 3.7.2. Estimation of Tumor Parameters

##### Tumor Volume Progression

**Figure 8:**
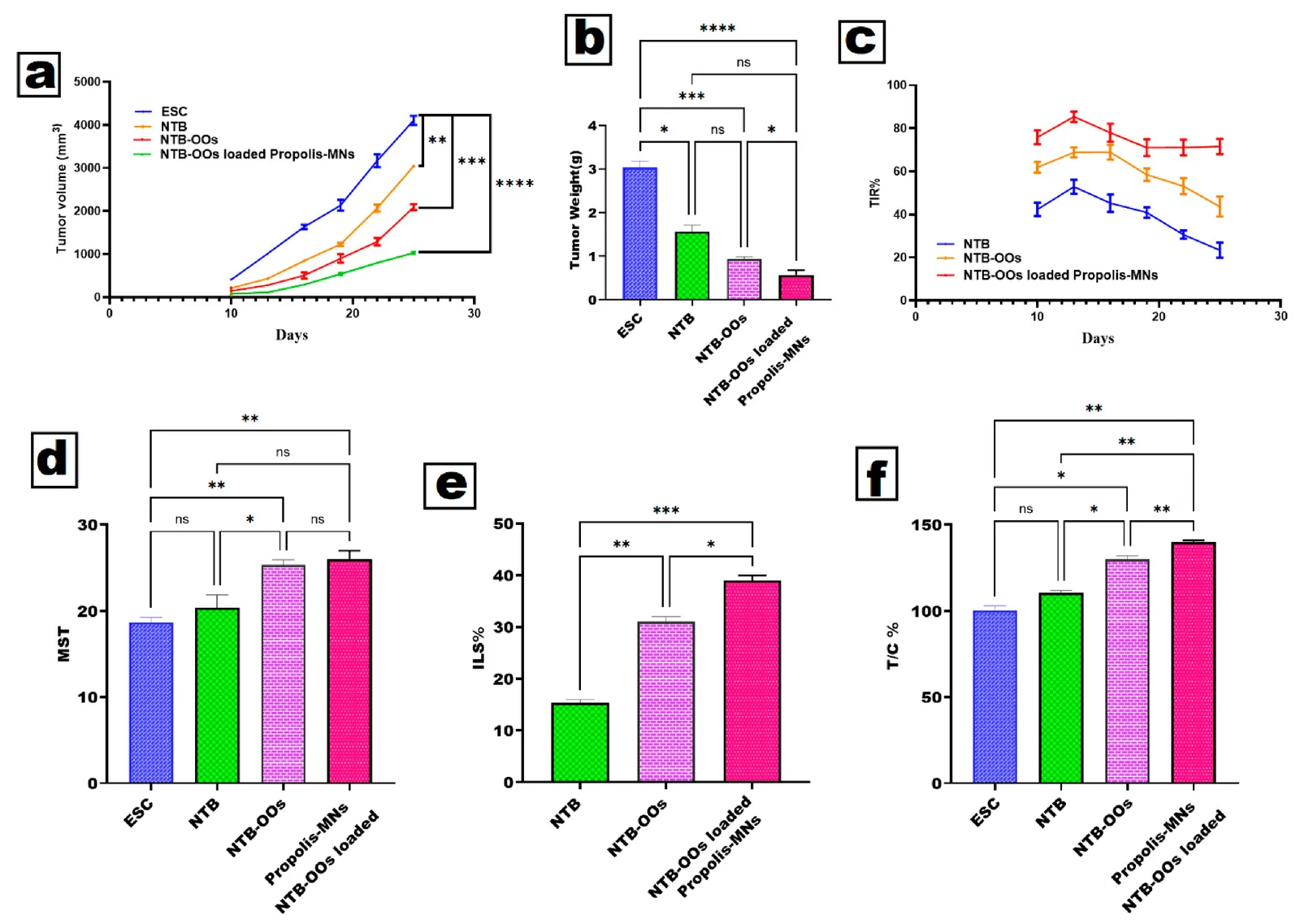
In vivo antitumor efficacy of different neratinib formulations in Ehrlich solid carcinoma-bearing mice; (**a**) Tumor volume (mm^3^) measured at the end of treatment, (**b**) Tumor weight (g) at the end of the experimental period, (**c**) Tumor inhibition rate (TIR%), (**d**) mean survival time (MST), (**e**) increase in life span (ILS%), and (**f**) T/C% of mice treated groups compared with the ESC control. Data are expressed as mean ± SD (n = 3). * *p* < 0.05, ** *p* < 0.01,*** *p* < 0.001, **** *p* < 0.0001, and ns (not significant) versus the control group. Data were expressed as Mean ± SD (n = 10).

As shown in Figure 8a, the untreated ESC group demonstrated continuous and rapid tumor growth during the whole experiment, eventually leading to the largest tumor volume at the end of the treatment period. This confirms the successful induction of tumors and the aggressive progression of tumors without therapy. In contrast, the ESC group showed a significant increase in tumor volume progression, whereas all NTB-treated groups showed a significant decrease.

The free NTB showed intrinsic antitumor activity with a moderate reduction in tumor growth. This effect is thought to be related to the irreversible pan-HER tyrosine kinase inhibition properties of neratinib. NTB inhibits tumor cell proliferation and survival by blocking HER-family signaling pathways (including EGFR/HER1 and HER2-related pathways). In previous preclinical studies, NTB has been shown to potently inhibit proliferation and downstream signaling in HER2-positive preclinical breast cancer cell models. The encapsulation of NTB in *Ocimum* oil nanovesicles enhanced its antitumor properties, as the NTB-OOs exhibited a higher tumor growth inhibition than free NTB. The enhancement might be related to increased local drug availability, improved interaction with biological membranes, vesicle-mediated drug delivery, and increased NTB solubilization [94]. The propolis microneedles loaded with NTB-OOs exhibited the strongest tumor-suppressive effect, with a significantly lower final tumor volume compared with all other groups. These findings suggest that combining dissolving microneedles with nanovesicular delivery improved the local therapeutic effect of NTB.

##### Tumor Weight Assessment

The tumor weight was also consistent with the tumor-volume results (Figure 8b). The tumor weight of the untreated ESC group was the highest, at 3 g. Conversely, the tumor weight of the free NTB group decreased to about 1.5 g. The tumor weight of the propolis microneedle group loaded with NTB-OOs was the lowest (approximately 0.5 g) and increased to approximately 0.9 g after NTB-OO administration. The progressive reduction in tumor weight evidenced the formulation-dependent antitumor response. The superiority of NTB-OOs over free NTB was demonstrated by the nanovesicular system’s enhancement of NTB delivery and exposure to tumors [5]. The further reduction by the loaded NTB-OOs microneedles is ascribed to the ability of the microneedles to bypass the stratum corneum, create microchannels, and enhance the transdermal deposition of NTB-OOs near the tumor-bearing tissue. Recently, it has been shown that microneedle-based cancer delivery systems can enhance local drug delivery by creating transient skin pathways and improving drug penetration into targeted tissues.

##### Tumor Inhibition Rate

The tumor inhibition rate (TIR%) during the treatment period is shown in Figure 8c. Free NTB showed the lowest TIR%, indicating partial inhibition of tumor growth. This confirms the enhanced antitumor activity of NTB due to nanoencapsulation, as the NTB-OOs showed higher TIR% values than free NTB. The TIR% of the NTB-OOs-loaded propolis microneedles was highest (estimated at 75–85% during treatment) and was significantly higher than the other formulations at the end of the experiment. This increase in TIR% indicates a successful suppression of tumour growth. The results are in agreement with the tumor-volume and tumor-weight data and confirm the order of efficacy, as NTB-OOs-loaded propolis-MNs are better than free NTB and untreated ESC. The enhanced TIR% in the microneedle group may be due to the combined effects of microneedle-assisted administration, nanovesicular NTB delivery, and NTB’s pharmacological efficacy. Another recent study on NTB-loaded solid lipid nanoparticles in dissolvable MNs also reported improved transdermal breast cancer therapy, supporting the strategy of combining neratinib nanocarriers with dissolving MNs.

##### Mean Survival Time

Figure 8d shows that the mean survival time of the treated groups increased progressively. The lowest MST was observed in the ESC group (approximately 19 days), and free NTB increased MST to approximately 21 days. NTB-OOs further prolonged MST to about 25 days, with the longest MST observed with NTB-OO-loaded propolis microneedles at about 26.5 days. This improvement in MST was associated with tumor suppression and provided evidence of prolonged survival. The significant improvement in MST for NTB-OOs and NTB-OO-loaded microneedles suggests that therapeutic efficacy was directly influenced by enhanced NTB delivery. Nanoparticle-based anticancer systems have been shown to improve survival, reduce tumor burden, and increase drug bioavailability relative to free agents, thus supporting the survival benefit of nanocarrier-based treatment. Previously, it has been reported that propolis nanoparticle treatment can inhibit the growth of Ehrlich ascites carcinoma and increase lifespan compared to free propolis [142,143].

##### Increase in Life Span

The percentage increase in life span, ILS%, is shown in Figure 8e. Free NTB resulted in a modest ILS% of about 10.5%, indicating only a limited survival benefit compared to the untreated ESC group. The ILS% was increased to ca. 31.6% by NTB-OOs, and the ILS% increased to ca. 39.5% by NTB-OOs-loaded propolis microneedles. The results presented showed that nanoformulation significantly enhanced the survival benefit of NTB. The enhanced ILS% of NTB-OOs can be attributed to the improved drug solubilization and vesicular delivery. The further enhancement in ILS% of NTB-OOs-loaded microneedles is attributed to the improved local drug deposition and sustained delivery through the skin [1,144]. The most used criteria for antitumor-efficacy evaluation are an ILS% of >25% and a T/C% of >125%, which indicate significant antitumor activity. Therefore, the survival of NTB-OOs and NTB-OOs-loaded propolis microneedles was significantly increased, whereas free NTB did not reach this level.

##### T/C%

The T/C% represents the survival of treated groups relative to the ESC control and is shown in Figure 8f. The ESC group was set to 100%, and free NTB was raised to T/C% to about 110.5%. The T/C% was increased to ~131.6% by NTB-OOs, and the highest T/C% was observed in propolis microneedles loaded with NTB-OOs at ~139.5%. These results are consistent with the ILS% results and provide additional evidence that NTB-OOs and NTB-OOs-loaded microneedles conferred significant survival benefits by the NCI-related criteria. The optimized NTB-OOs/P-MNs formulation showed the best antitumor efficacy, as indicated by the highest T/C% of the microneedle group.

The augmented activity of NTB-OOs may also be attributed to the biological properties of *Ocimum* oil. *Ocimum basilicum* and its phytoconstituents have been reported to exhibit anticancer activity in different in vitro and in vivo models, including breast cancer cell lines. The review on the anticancer activity of *basil* described the ability to modulate proliferation and tumor-related pathways. In addition, a study reported cytotoxicity of *Ocimum basilicum* essential oil against MCF-7 cells [2,145]. Thus, *Ocimum* oil can serve as both a vesicular component and a supportive bioactive oil phase to enhance drug delivery and membrane interactions. Propolis can also improve the therapeutic efficacy of microneedle formulation. The flavonoids and phenolic compounds in propolis have been reported to inhibit cancer cell proliferation, induce apoptosis, and modulate tumor-related signaling pathways. In the current system, propolis likely had a dual role, being a part of the polymeric matrix of the microneedles and exerting supportive anticancer or anti-inflammatory effects at the tumor site. In addition, previous studies have shown that propolis-derived nanoparticles improve the anti-tumor response and lifespan of Ehrlich tumor-bearing mice, confirming the importance of delivery systems containing propolis in cancer therapy. Overall, the results confirm that the antitumor efficacy of NTB was strongly dependent on the delivery system. Free NTB showed moderate antitumor activity, but its effect was limited, probably due to insufficient local delivery and limited skin permeation. Loading NTB into *Ocimum* oil nanovesicles improved tumor suppression, reduced tumor weight, increased TIR%, and enhanced survival parameters. The best therapeutic outcome was obtained after loading NTB-OOs into PVA/PVP–propolis dissolving microneedles. This formulation combined the anticancer activity of NTB, the vesicular delivery advantage of *Ocimum* oil nanovesicles, the biological support of propolis, and the physical penetration ability of microneedles. Thus, the optimized NTB-OOs-loaded propolis microneedles achieved the greatest reductions in tumor volume and weight, the highest TIR%, the longest MST, and the greatest ILS% and T/C%. These findings support the potential of NTB-OOs/P-MNs as an effective localized transdermal platform for breast cancer therapy.

#### 3.7.3. Assessment of Inflammatory Biomarkers and Oxidative Stress Assessment

**Figure 9:**
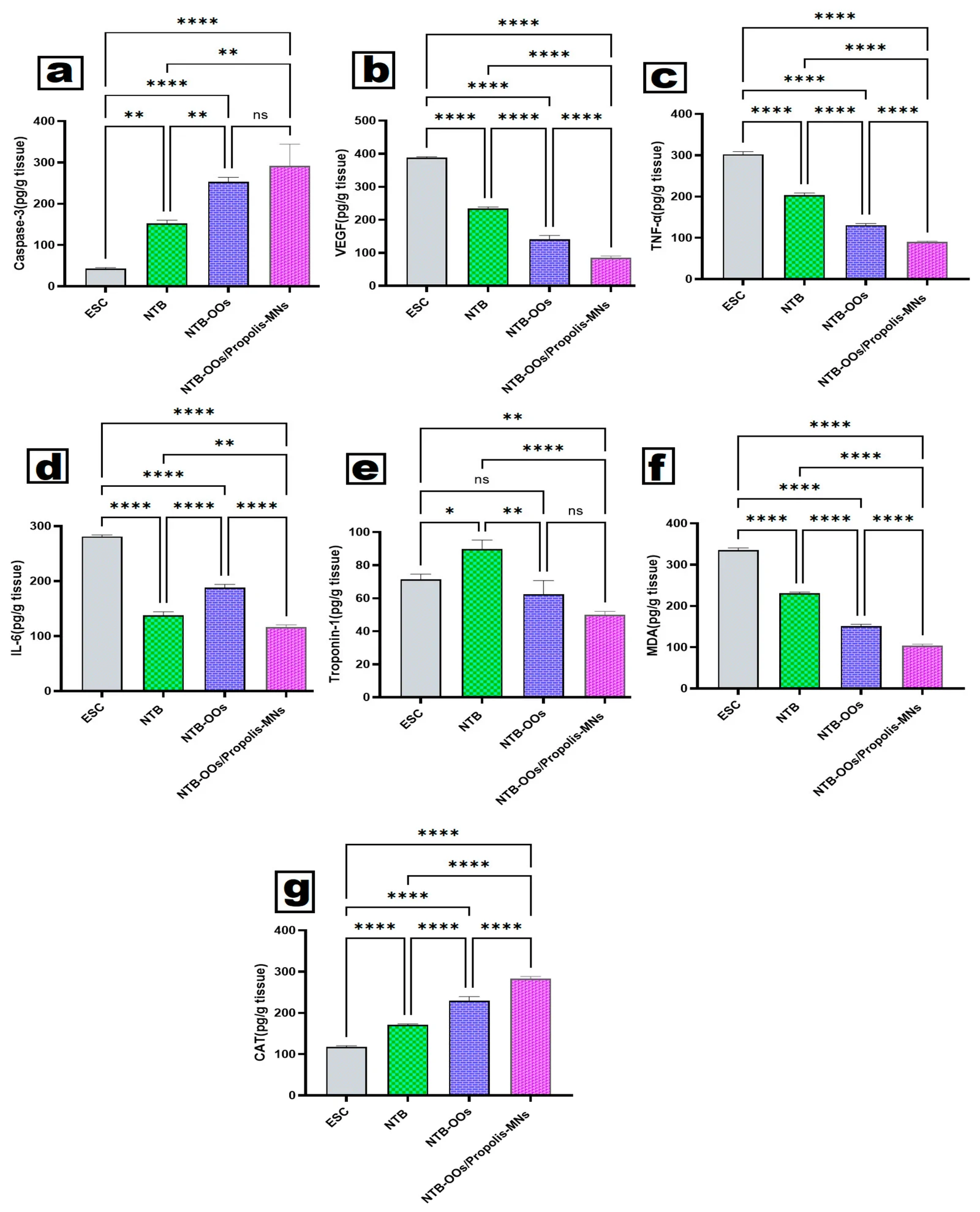
Effects of different treatments on inflammatory biomarkers measured by ELISA: (**a**) Caspase-3, (**b**) VEGF, (**c**) TNF-α, (**d**) IL-6, (**e**) Troponin-1, (**f**) MDA, and (**g**) CAT. * *p* < 0.05, ** *p* < 0.01, **** *p* < 0.0001, and ns (not significant) versus the control group. Data were expressed as Mean ± SD, n = 3.

Figure 9 evaluates the biochemical response of tumor-bearing mice after treatment with free NTB, NTB-OOs, and NTB-OOs-loaded PVA/PVP–propolis microneedles. Apoptosis occurred via caspase-3, angiogenesis via VEGF, inflammation via TNF-α and IL-6, cardiac safety via troponin-I, and oxidative stress/antioxidant defense via MDA and CAT. The results show that the untreated ESC group exhibited low apoptotic activity, elevated levels of angiogenic and inflammatory biomarkers, high oxidative stress, and weak antioxidant defense. Treatment shifted this profile toward a more favorable antitumor state, with the strongest improvement generally observed in the NTB-OOs-loaded propolis microneedle group.

Caspase-3 levels were markedly increased after treatment, as shown in Figure 9a. The ESC group had the lowest caspase-3 level, at about 40 pg/g tissue, suggesting that, in untreated tumors, apoptotic activity is low. Free NTB increased caspase-3 to about 150 pg/g tissue and NTB-OOs to about 250 pg/g tissue. The NTB-OOs/propolis-MNs group had the highest caspase-3 level, approximately 290 pg/g tissue. This increase suggests that the apoptosis of tumor cells has been increased. Caspase-3, a major executioner caspase, participates in nuclear fragmentation, chromatin condensation, and apoptotic cell death. Thus, its elevation indicates that the treatment promotes programmed tumor-cell death, in particular the microneedle-loaded nanovesicular formulation. This is in line with the known anticancer mechanism of NTB, i.e., the inhibition of HER-family signaling pathways involved in cell proliferation and survival. Previous studies have shown that NTB can inhibit HER1/HER2/HER4 signaling and exert anticancer activity in breast cancer models. Improved drug availability within cells is indicated by increased caspase-3 activation in NTB-OOs and NTB-OOs/propolis-MNs compared to free NTB. Microneedles can improve local delivery by overcoming the skin barrier and delivering NTB-OOs more effectively near the tumor site, and the nanovesicular system can improve NTB solubilization and tissue interaction [146,147].

The untreated ESC group showed a significant increase in VEGF, as shown in Figure 9b, estimated at 385 pg/g tissue, suggesting high angiogenic activity. VEGF decreased to approximately 230 pg/g tissue after free NTB, 135 pg/g tissue after NTB-OOs, and to approximately 80 pg/g tissue after NTB-OOs/propolis-MNs. VEGF is an important pro-angiogenic factor involved in tumor vascularization. High levels of VEGF help tumors grow by stimulating new blood vessels to grow to provide them with oxygen and nutrients. Hence, the reduction of VEGF levels after treatment was statistically significant and indicative of inhibition of tumor angiogenesis. Previous studies on breast cancer have demonstrated that VEGF is a major regulator of tumor progression and angiogenesis. The group of NTB-OOs/propolis-MNs showed the most significant VEGF suppression. These results are consistent with the tumor-volume and tumor-weight results, indicating the most potent antitumor effect of this formulation. This means that the microneedle-loaded formulation not only induced apoptosis but also reduced angiogenic support for tumor growth.

The concentration of TNF-α was highest in the ESC group, at about 300 pg/g in tissue, as shown in Figure 9c. This concentration confirmed the presence of a strong inflammatory tumor microenvironment. The free NTB reduced the TNF-α level to approximately 200 pg/g of tissue, and the NTB-OOs to approximately 125 pg/g of tissue. NTB-OOs/propolis-MNs achieved the greatest reduction (~90 pg/g tissue).

IL-6 showed a similar reduction overall compared to ESC. The highest level of IL-6 was found in the ESC group (ca. 275–280 pg/g tissue). NTB-OOs produced the lowest IL-6 value (~115 pg/g tissue), while NTB-OOs/propolis-MNs produced the highest IL-6 value (~180–190 pg/g tissue), which was higher than free NTB but lower than ESC. Free NTB decreased IL-6 to ~135 pg/g tissue. TNF-α and IL-6 are important inflammatory cytokines involved in cancer progression, tumor cell survival, angiogenesis, invasion, and resistance to some treatments. The tumor microenvironment is persistently inflamed, and cytokines like TNF-α and IL-6 contribute to tumorigenesis. The reduction in TNF-α and IL-6 indicated that NTB treatment reduced tumor-associated inflammation. The greater reduction of the microneedle-loaded formulation may be due to the presence of propolis, which has been documented to possess anti-inflammatory and anticancer properties and better local NTB delivery. Previous reviews have shown that propolis and its polyphenolic constituents have the potential to suppress pro-inflammatory mediators such as TNF-α and IL-6 and potentially exert anticancer effects via inducing apoptosis and modulating tumor-related signaling pathways. The IL-6 profile deserves careful interpretation because NTB-OOs reduced IL-6 compared with ESC, but not as strongly as free NTB. This may indicate that the IL-6 response does not always directly parallel tumor volume reduction, or that the oil-vesicular system may elicit a formulation-specific immune response. However, the NTB-OOs/propolis-MNs group showed the lowest IL-6 level, confirming that the final optimized formulation achieved the best anti-inflammatory balance.

Troponin-I was used as a cardiac safety marker, as shown in Figure 9e. The ESC group showed a value of around 70 pg/g tissue. Free NTB increased troponin-I to approximately 90 pg/g tissue, suggesting some treatment-associated cardiac stress. In contrast, NTB-OOs reduced troponin-I to around 60 pg/g tissue, and NTB-OOs/propolis-MNs reduced it further to approximately 50 pg/g tissue. Troponins are widely used as sensitive biomarkers of myocardial injury and cardiotoxicity in cancer therapy. HER2-targeted treatments are known to require cardiac monitoring because cardiotoxicity is a recognized concern in breast cancer treatment, and troponin elevation can indicate early myocardial damage. The lower troponin-I values in NTB-OOs and NTB-OOs/propolis-MNs suggest that the optimized delivery systems may reduce systemic cardiac exposure or oxidative cardiac stress compared with free NTB. This is an important advantage because an effective anticancer formulation should ideally improve tumor suppression without increasing cardiac toxicity [50,66].

MDA was markedly elevated in the ESC group, approximately 335 pg/g tissue, indicating high lipid peroxidation and oxidative stress, as shown in Figure 9f. Free NTB decreased MDA to approximately 230 pg/g tissue; NTB-OOs to 150 pg/g tissue and NTB-OOs/propolis-MNs to 100 pg/g tissue. However, the CAT activity showed the opposite trend. The ESC group had the lowest level of CAT (~115 pg/g tissue), indicating the impairment of the antioxidant defense. Free NTB increased the level of CAT to about 170 pg/g tissue, and NTB-OOs to 230 pg/g tissue and NTB-OOs/propolis-MNs to 280 pg/g tissue, as shown in Figure 9g. Catalase is an important antioxidant enzyme involved in the detoxification of hydrogen peroxide. MDA is a common marker of lipid peroxidation and oxidative stress. Therefore, the decrease in MDA together with the increase in CAT indicates the restoration of redox balance [1,2].

The strongest antioxidant improvement in the NTB-OOs/propolis-MNs group may be attributed to several factors. First, better tumor suppression reduces oxidative burden. Second, *Ocimum* oil contains bioactive constituents with reported antioxidant and anti-inflammatory effects. Third, propolis contains flavonoids and phenolic compounds with antioxidant activity. Previous studies reported antioxidant and anti-inflammatory activities of *Ocimum basilicum* essential oil and propolis, supporting their possible contribution to the improved oxidative-stress profile in the present formulation. Taken together, the ELISA findings confirm that the antitumor efficacy of NTB was improved by formulation into *Ocimum* oil nanovesicles and further enhanced after incorporation into PVA/PVP–propolis dissolving microneedles. The optimized NTB-OOs/propolis-MNs formulation produced the most favorable biomarker profile; the increased caspase-3, decreased VEGF, TNF-α, IL-6, troponin-I, and MDA, and increased CAT. This indicates that the formulation acted through multiple complementary mechanisms: induction of apoptosis, inhibition of angiogenesis, suppression of tumor-associated inflammation, reduction in oxidative stress, improvement of antioxidant defense, and reduction in cardiac injury markers. These effects are consistent with the previously observed reductions in tumor volume and tumor weight and support the selection of NTB-OOs-loaded propolis microneedles as the most effective and biologically favorable formulation. The biomarker analysis suggested that NTB-OOs-loaded PVA/PVP–propolis microneedles showed a better antitumor effect than free NTB and NTB-OOs by simultaneously inducing apoptosis, suppressing angiogenesis and inflammatory signaling, diminishing oxidative stress, boosting antioxidant defense, and reducing cardiac damage.

#### 3.7.4. Liver Function Tests (LFTs), Kidney Function Tests

Table 9 shows that the untreated Ehrlich solid carcinoma (ESC) model induced severe hepatorenal injury, as evidenced by increased serum levels of AST, ALT, ALP, total bilirubin, urea, creatinine, and uric acid, as determined by hepatic and renal biomarker assessment. Increased AST and ALT are usually indicative of hepatocellular damage, and increased ALP and bilirubin may be indicative of biliary or mixed hepatic dysfunction. Likewise, increased urea and creatinine are classic signs of renal impairment, and increased uric acid may be indicative of impaired purine metabolism, oxidative stress, and decreased renal clearance. The unadulterated NTB gel treatment showed modest improvement in liver and kidney biomarkers compared with the ESC model. AST, ALT, ALP, and total bilirubin decreased approximately 13.3%, 15.7%, 12.6%, and 16.9%, respectively, and urea, creatinine, and uric acid decreased approximately 13.6%, 14.6%, and 18.8%, respectively. The partial improvement may be due to NTB’s antitumor effect as a pan-HER tyrosine kinase inhibitor with irreversible binding, while the substandard performance of the plain gel may be attributable to NTB’s poor aqueous solubility and limited permeability, which hinder effective transdermal delivery. NTB is associated with clinically relevant elevations in liver enzymes and requires monitoring of liver function during therapy, highlighting the need to evaluate the hepatorenal safety of NTB-containing formulations [2]. Conversely, greater enhancements were observed in hepatic and renal markers with the NTB-OOs gel. Compared with the ESC model, AST, ALT, ALP and total bilirubin were decreased by approximately 29.6%, 33.1%, 27.3% and 34.8%, respectively. Approximately a 29.3%, 31.5%, and 37.5% reduction in urea, creatinine, and uric acid levels, respectively. The enhancement indicates that encapsulation of NTB in *Ocimum basilicum* oil nanovesicles enhanced its biological efficacy and reduced systemic biochemical stress. This improvement can be attributed to improved drug solubilization, nanoscale vesicular entrapment, improved interaction with the skin barrier, and sustained release from lipid-based nanovesicles. In addition, *Ocimum basilicum* contains bioactive volatile and phenolic compounds with antioxidant and hepatorenal protective activities, which could reduce oxidative stress-induced liver and kidney damage. The greatest protective effect was observed in the NTB-OOs/P-MNs group. This formulation resulted in decreases in AST, ALT, ALP, and total bilirubin by about 36.4%, 44.0%, 36.3%, and 46.1%, respectively, compared with the ESC model. Renal biomarkers showed a similar pattern, with urea, creatinine, and uric acid decreased by approximately 40.5%, 41.6%, and 50.0%, respectively. The enhanced performance of NTB-OOs/P-MNs arises from the synergy of nanovesicular drug loading and microneedle transdermal delivery. Microneedles penetrate the stratum corneum and create transient microchannels to deliver drug-loaded nanovesicles into the viable epidermal and dermal layers, enhancing drug permeation and maintaining systemic absorption, while overcoming the limitations of conventional topical gels. Previous studies have shown that the transdermal delivery of nanoparticles can be enhanced by using microneedles for the delivery of nanoparticles in order to increase the efficacy of therapeutic delivery. The integration of PVA/PVP and propolis in the dissolving microneedle matrix could have benefited the safety profile. PVA and PVP are the most widely used hydrophilic polymers in dissolving microneedles due to their ability to hydrate quickly, dissolve, and rapidly release drugs after insertion into the skin. The extract is rich in flavonoids and phenolic compounds and exhibits antioxidant, anti-inflammatory, hepatoprotective, and nephroprotective effects on experimental toxicity models. Thus, the better reduction in liver and kidney biomarkers in the NTB-OOs/P-MNs group could be attributed to a synergistic effect derived from increased NTB delivery, slow release of nanovesicles, decreased oxidative/inflammatory stress, and the intrinsic protective effects of *Ocimum basilicum* oil and propolis. The results showed improvement of hepatorenal biochemical status in dependence on formulation in the order of NTB-OOs/P-MNs > NTB-OOs gel > NTB gel > ESC model. The trend is consistent with the hypothesis that nanovesicular encapsulation and microneedle-assisted delivery of NTB increases its therapeutic index by improving its delivery efficiency and mitigating hepatorenal stress associated with tumor burden. However, the lack of a normal healthy control group in this table means that the results should be interpreted as an improvement in ESC-induced biochemical alterations rather than complete normalization.

#### 3.7.5. Effect of Different Formulations on Lipid Profile

Table 10 shows that the untreated ESC model exhibited a significant dyslipidemic pattern, with increases in TG, TC, LDL-C, and VLDL-C, and a decrease in HDL-C. This phenomenon may be attributed to tumor-associated metabolic reprogramming, systemic inflammation, oxidative stress, and altered lipid utilization by rapidly proliferating tumor cells. Changes in lipid metabolism are tightly linked to cancer progression. Tumor proliferation, inflammation, and disease progression are related to the levels of TC, TG, LDL-C, and HDL-C. NTB gel treatment showed a moderate improvement in the lipid profile in the ESC model. Triglycerides, total cholesterol, LDL cholesterol, and VLDL cholesterol were reduced by about 13.9%, 13.8%, 22.2%, and 13.8%, respectively, and HDL cholesterol was increased by about 18.2%. This improvement may be related to the antitumor efficacy of NTB, which can relieve tumor-associated metabolic stress and inflammatory burden. But the conventional gel preparation only provided a partial correction, possibly due to poor skin permeation and insufficient aqueous solubility of neratinib, which might limit drug availability at the therapeutic site. A marked improvement was observed after the application of NTB-OOs gel. TG, TC, VLDL-C and LDL-C decreased by approximately 28.9%, 27.7%, 44.8% and 28.9%, respectively, compared with the ESC model. HDL-C increased by approximately 38.8%. The use of NTB in *Ocimum basilicum* oil nanovesicles enhanced the biological efficacy of the drug. This improvement is due to the better solubilization of the drug, nanoscaled vesicular encapsulation, increased interaction with the skin surface, and prolonged release of the drug. Furthermore, previous studies have shown that *Ocimum basilicum* has antioxidant and hypolipidemic properties, such as decreasing TC, TG, and LDL-C, which could be useful in improving lipid metabolism and reducing oxidative stress. The greatest effect on lipid modulation was seen in the NTB-OOs/P-MNs group. The formulation decreased TG, TC, LDL-C and VLDL-C by approximately 39.9%, 38.5%, 62.4% and 40.0%, respectively, compared with the ESC model, and increased HDL-C by approximately 54.5%. This group exhibited enhanced efficacy owing to the synergistic combination of three formulation benefits: nanovesicular encapsulation of NTB, the lipid-modulating and antioxidant properties of *Ocimum basilicum* oil, and microneedle-assisted transdermal delivery. Dissolving microneedles can penetrate the stratum corneum to form transient microchannels to enable the transport of drug-loaded nanoparticles into the viable epidermis and dermis and enhance drug availability in the systemic circulation. The use of PVA/PVP–propolis microneedles may improve the lipid profile. Propolis contains flavonoids and phenolic compounds that have antioxidant and anti-inflammatory effects. Previous studies show that propolis supplementation may significantly reduce triglyceride levels and increase HDL cholesterol levels. The marked increase in HDL-C and reduction in atherogenic lipids in the NTB-OOs/P-MNs group may suggest the synergistic effect of improved NTB delivery, reduction in tumor-associated inflammation, enhanced oxidative balance, and beneficial biological activities of *O. basilicum* oil and propolis.

#### 3.7.6. Quantitative Real-Time Polymerase Chain Reaction (qRT-PCR) Assessment

**Figure 10:**
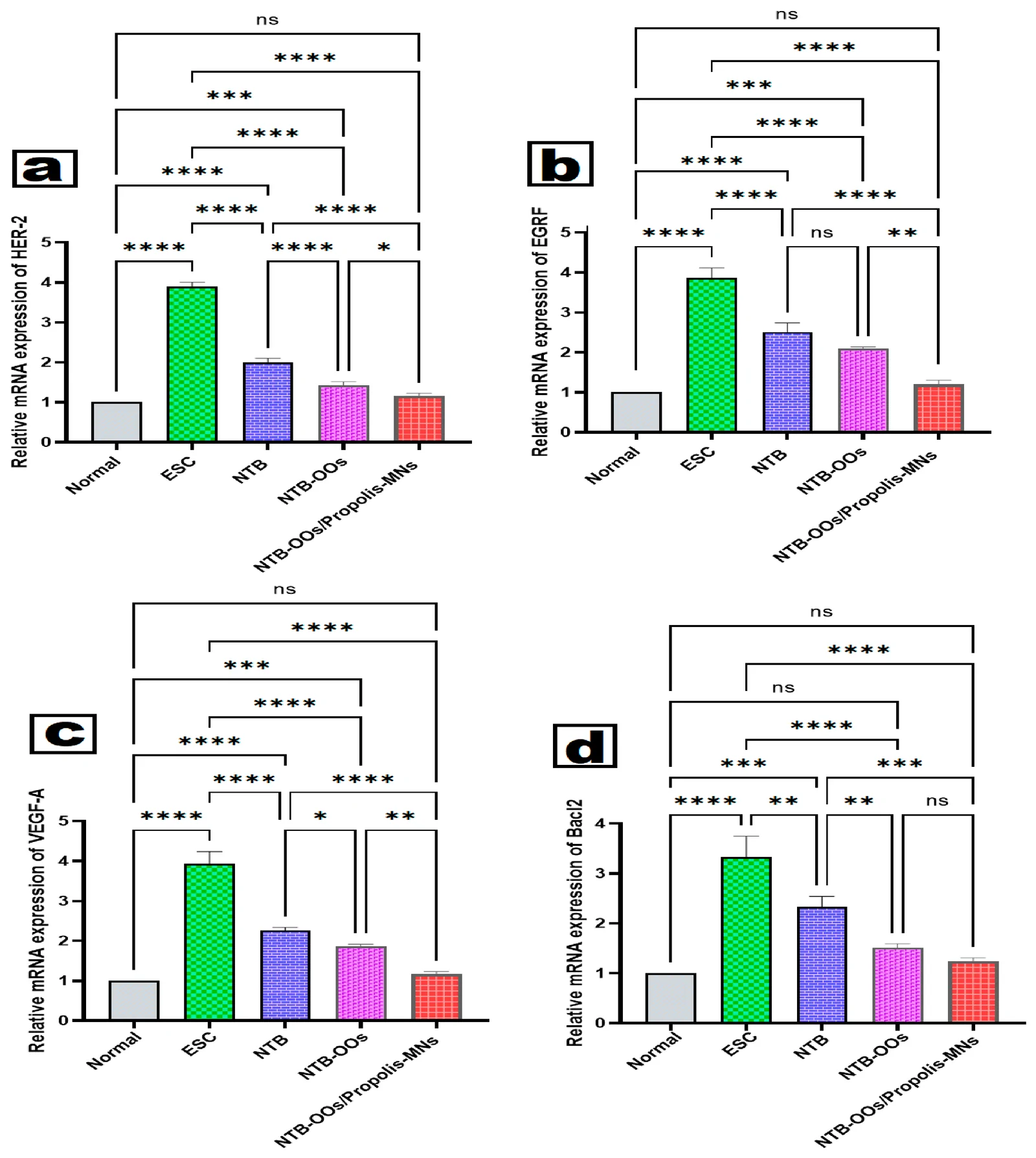
Effect of different treatments on the relative mRNA expression of tumor-associated genes in Ehrlich solid carcinoma-bearing mice, demonstrating the relative transcription levels of HER2 (**a**), EGFR (**b**), VEGF-A (**c**), and Bcl2 (**d**). Data are expressed as mean ± SD (n = 3). * *p* < 0.05, ** *p* < 0.01, *** *p* < 0.001, **** *p* < 0.0001, and ns (not significant) versus the control group.

Figure 10 shows the effects of different NTB formulations on the relative mRNA expression levels of HER2, EGFR, VEGF-A, and Bcl-2 in tumor tissue. These genes were selected as they are indicative of key processes in breast tumor progression: HER-2 and EGFR are markers of oncogenic growth signaling, VEGF-A is a marker of angiogenesis, and Bcl-2 is a marker of anti-apoptotic survival activity. All four genes were significantly upregulated in the ESC group compared to the normal group, and their expression was markedly decreased after treatment with NTB, NTB-OOs, and especially NTB-OOs-loaded PVA/PVP–propolis microneedles.

The ESC group had significantly higher HER-2 mRNA expression, approximately 3.8–4.0 times that of the normal group, as shown in Figure 10a. This confirms activation of HER family oncogenic signaling in tumor-bearing mice. HER-2 overexpression has been correlated significantly with aggressive features of breast cancer through activation of downstream proliferative and survival pathways such as PI3K/AKT and MAPK. NTB is an irreversible pan-HER tyrosine kinase inhibitor that specifically inhibits HER1/EGFR, HER2, and HER4, causing receptor autophosphorylation and downstream signaling. Earlier preclinical studies demonstrated that NTB potently suppressed the growth of HER2-positive breast cancer cells and downstream signaling pathways. Free NTB reduced HER-2 expression by ~2-fold, demonstrating neratinib’s ability to inhibit HER-2-mediated signaling. NTB-OOs showed greater reduction, downregulating HER-2 expression to about 1.3–1.5-fold. In contrast, NTB-OOs loaded with propolis microneedles downregulated HER-2 expression to near-normal levels, by about 1.1-fold. This illustrates that the nanovesicular loading system of microneedles can effectively inhibit HER-2 expression, which is probably due to the enhanced local deposition and sustained release of NTB at the tumor site [138].

EGFR expression followed a similar pattern, as shown in Figure 10b. The ESC group showed a significant increase in EGFR mRNA expression, approximately 3.8–4.0-fold compared with normal tissue. EGFR is important because it supports tumor growth, migration, survival, and resistance mechanisms. NTB, as part of its pan-HER activity, directly inhibits EGFR/HER1, and the reduction in EGFR expression after treatment supports NTB’s targeted mechanism of action. NTB has been shown to inhibit ligand-phosphorylated HER2 and EGFR activity and to block downstream MAPK signaling. The level of EGFR expression was reduced by ~2.4–2.6-fold with Free NTB and by ~2.0–2.1-fold with NTB-OOs. The greatest reduction was observed in the NTB-OOs/propolis-MNs group, where EGFR expression was reduced to approximately 1.1–1.2-fold, a level quite similar to that of the normal group. These results indicate that incorporating NTB-OOs into dissolvable microneedles enhanced drug delivery and resulted in a more effective reduction in EGFR expression than free NTB or NTB-OOs gel.

VEGF-A was upregulated in the ESC group, approximately 3.8–4.0-fold higher than in normal tissue, as shown in Figure 10c. VEGF-A is an important angiogenic factor responsible for the formation of new blood vessels, which allows tumors to acquire the oxygen and nutrients necessary for growth and progression. Therefore, the high expression of VEGF-A in the ESC group is associated with active tumor angiogenesis. The treatment downregulated VEGF-A expression in a formulation-dependent manner. Free NTB reduced VEGF-A expression by approximately 2.2–2.3-fold, NTB-OOs by approximately 1.8-fold, and NTB-OOs/propolis-MNs normalized it to near-normal levels, approximately 1.1-fold. This demonstrates that the optimized microneedle formulation possesses potent anti-angiogenic activity. This finding is consistent with the ELISA results, which demonstrated a decrease in VEGF protein levels following treatment. Previous studies have shown that NTB is able to downregulate VEGF and has anti-angiogenic effects, confirming our present findings on VEGF-A mRNA. The decrease in VEGF-A may be due to blockade of HER2/EGFR signaling, as HER signaling can activate angiogenic pathways. The significant reduction of VEGF-A in the NTB-OOs/propolis-MNs group suggests that the formulation inhibited the signaling pathways of tumor cell proliferation and the angiogenic support required for tumor growth.

Bcl-2 expression was higher in the ESC group (about 3.2 to 3.4 times that of normal tissue) as shown in Figure 10d. Bcl-2 is an anti-apoptotic protein that inhibits mitochondrial apoptosis and promotes the survival of tumor cells under stress conditions. Bcl-2 upregulation in the ESC group indicates enhanced survival potential and resistance to apoptosis in tumor cells. NTB-OOs further decreased Bcl-2 to 1.5-fold, while free NTB decreased it to 2.2–2.4-fold. The lowest expression of Bcl-2 was found with NTB-OOs/propolis-MNs, approximately 1.2-fold, which was almost similar to the normal group. The decrease in Bcl-2 promotes apoptosis and correlates with ELISA data showing increased caspase-3 levels upon treatment. The downregulation of Bcl-2 and the upregulation of caspase-3 represent a transition from tumor cell survival to apoptosis.

This result is consistent with previous studies showing that the best antitumor treatment for ESC models is usually associated with the inhibition of anti-apoptotic markers, such as Bcl-2, and the induction of apoptotic mediators. Therapies that have been effective in solid Ehrlich carcinoma models have been demonstrated to affect markers of apoptosis and slow tumor progression. The increase in gene expression followed the following order: NTB-OOs/propolis-MNs > NTB-OOs > free NTB > SEC. This order confirmed that the therapeutic effect depended not only on the pharmacological activity of NTB but also on the delivery system. Free NTB significantly inhibited the expression of HER-2, EGFR, VEGF-A and Bcl-2. This could be because of limited local delivery and insufficient penetration when NTB is used unbound. NTB-OOs showed greater downregulation, suggesting that Ocimum oil nanovesicles enhanced NTB solubilization, tissue interaction, and local availability. *Ocimum* species and their bioactive constituents have shown anticancer potential, including modulation of apoptosis and oxidative/inflammatory pathways, which may improve the efficacy of the NTB-loaded vesicles. The best gene-expression profile was obtained by NTB-OOs-loaded PVA/PVP–propolis microneedles. This phenomenon could be ascribed to the dual delivery mechanism where microneedles bypass the stratum corneum and create microchannels, while nanovesicles promote NTB dispersion and maintain localized drug availability. Recently, NTB-loaded solid lipid nanoparticles incorporated in dissolving microneedles have been identified as a promising transdermal delivery method for breast cancer therapy. Propolis might have also potentiated the therapeutic effect. Propolis has phenolic and flavonoid compounds that act as anticancer agents by inhibiting proliferation and angiogenesis, inducing apoptosis, and modulating tumor-associated signaling pathways. Thus, the propolis-loaded microneedle matrix may have enhanced the antitumor response biologically and mechanically. The molecular antitumor effect of NTB-OOs loaded PVA/PVP–propolis microneedles was the strongest according to the qRT-PCR analysis. The optimized formulation significantly downregulated HER-2 and EGFR, indicating the suppression of HER-family oncogenic signaling; reduced VEGF-A, indicating the inhibition of angiogenesis; and decreased Bcl-2, indicating the suppression of anti-apoptotic survival pathways. The molecular findings were consistent with the previously observed reductions in tumor volume and weight, VEGF protein levels, inflammatory cytokines, and oxidative stress, and an increase in caspase-3. The PCR results provided mechanistic evidence that the enhanced antitumor efficacy of NTB-OOs/propolis-MNs was associated with the simultaneous inhibition of tumor growth signaling, angiogenesis, and survival pathways with increased apoptosis. The qRT-PCR analysis showed that the optimized NTB-OOs/PVA-PVP–propolis microneedles exhibited the strongest molecular antitumor effect by downregulating the expression of HER-2, EGFR, VEGF-A and Bcl-2. The results confirm that the formulation enhanced NTB-induced inhibition of HER-family signaling, angiogenesis, and anti-apoptotic survival pathways, supporting its enhanced in vivo antitumor efficacy. A significant limitation of the present in vivo design was the absence of drug-free PVA/PVP microneedle and propolis-only control groups. Thus, the improved antitumor activity of NTB-OOs/P-MNs cannot be simply attributed to the propolis, polymeric microneedle matrix or the delivery mechanism. Thus, the current findings show the efficacy of the combined formulation globally, while the individual biological impact of each carrier component needs evaluation in properly controlled factorial experiments.

### 3.8. Histopathologic Analysis

**Figure 11:**
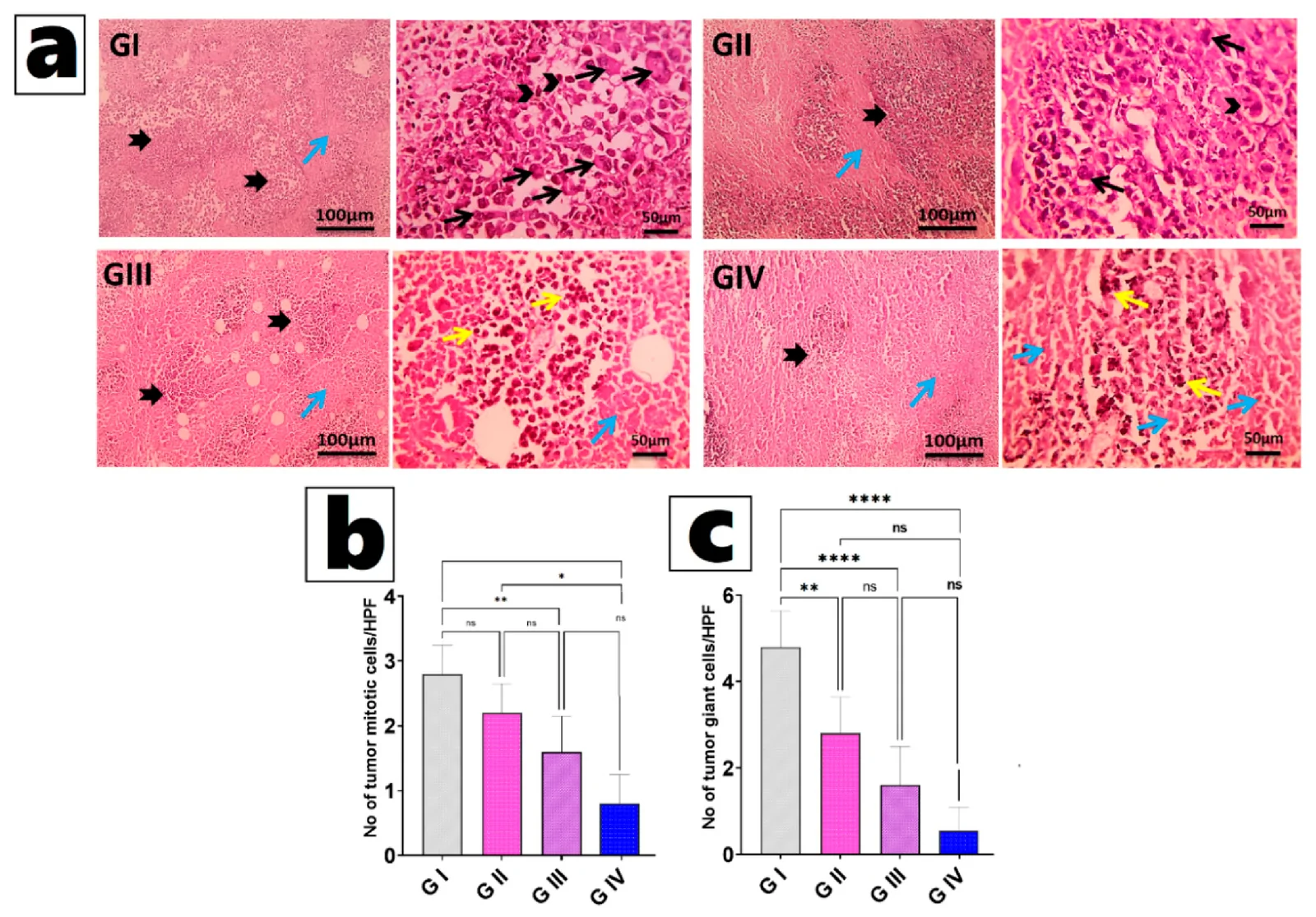
(**a**) Representative H&E-stained tumor sections from (GI) and treated groups (GII–GIV). GI showed 80% viable tumor tissue and 20% necrosis, while GII, GIII, and GIV showed 75%, 60%, and 30% viable areas with 25%, 40%, and 70% necrosis, respectively. Viable areas, necrosis, giant tumor cells, apoptotic cells, and mitotic figures are indicated by thick black arrows, blue arrows, thin black arrows, yellow arrows, and arrowheads, respectively. Images were captured at ×100, scale bar = 100 µm, and ×400, scale bar = 50 µm; (**b**) Quantitative analysis of the average number of mitotic cells, and (**c**) Quantitative analysis of the average number of giant tumor cells. Abbreviations: GI (ESC model, untreated), GII (Neratinib gel with 2% *w*/*w* HPMC), GIII (NTB-OOs in gel with 2% *w*/*w* HPMC), and GIV (NTB-OOs/PVA-PVP Propolis-MNs). Data are presented as mean ± SD (n = 3). * *p* < 0.05, ** *p* < 0.01, **** *p* < 0.0001 and ns (not significant).

Figure 11 shows the H&E-stained tumor sections, confirming a formulation-dependent anti-tumor response. The untreated ESC group exhibited the most aggressive histological features of the gastrointestinal tract, with roughly 80% viable tumor and 20% necrosis. The tumor tissue was highly cellular with clear areas of viable malignant cells, giant tumor cells, mitotic figures, and low necrosis. This means active tumor cell proliferation and insufficient spontaneous apoptosis. ESC is a frequently used experimental tumour model in breast cancer research. Tumors left untreated usually show a dense infiltrate of malignant cells, fast growth, and increased proliferative activity. Free NTB gel injection in GII resulted in a small decrease in the area of viable tumor to 75% and an increase in necrosis to 25%. Histologically, this modest improvement supports the hypothesis that NTB has intrinsic antitumor activity; however, its efficacy was limited when given as a conventional gel. This may be due to poor free NTB penetration through the dermis and limited accumulation of the drug in the tumor tissue. In GIII, NTB-OOs gel treatment significantly reduced the viable tumor area to 60% and increased the necrosis to 40%. These results indicate that incorporating NTB into *Ocimum* oil nanovesicles has enhanced its localized antitumor effect. The vesicular system may enable the solubilization of NTB, its interaction with membranes, and its delivery to tissues, thereby increasing damage to tumor cells compared with free NTB. The highest histopathological response was observed in GIV treated with NTB-OOs/PVA-PVP-propolis microneedles. In this cohort, only 30% of the tissue was viable tumor, with 70% necrotic. Necrotic areas were delineated, viable tumor areas decreased, and apoptotic characteristics increased, which meant that tumor cells were obliterated extensively. The results confirm that dissolving microneedles more effectively penetrate the stratum corneum and deliver NTB-OOs to the dermis or tumor site. A separate investigation on NTB further validated the utility of dissolvable microneedles loaded with NTB lipid nanoparticles as a potential transdermal approach for breast cancer therapy. Figure 11a confirms the following histological efficacy hierarchy: GIII precedes GIIII, GII, GI, and GIV. These results are consistent with those for tumor volume, tumor weight, ELISA, and PCR. The optimized NTB-OOs/propolis-MNs formulation showed the most serious damage to antitumor tissue. Figure 11b shows quantitative assessment of cells in mitosis. The mean number of mitotic cells in tumor sections is shown in Figure 11b. Mitotic figures are important histologic markers of tumor proliferation. A high mitotic count is a sign of increased tumor cell proliferation and increased malignancy. Mitotic activity is an important marker of tumor proliferation in breast cancer histopathology and is included in the tumor grading systems. The mitotic cell count was highest in the untreated GI group, suggesting increased tumor cell proliferation. The free NTB gel GII, applied topically, showed a moderate decrease in mitotic cells, indicating partial inhibition of tumor proliferation. NTB-OOs GIII showed a more pronounced decrease, indicating increased antiproliferative efficacy upon being loaded in nanovesicles. The lowest mitotic count was observed in GIV, indicating that the NTB-OOs/propolis-MNs were most effective at inhibiting mitotic division in tumor cells. The decrease in mitotic cells could be due to increased NTB delivery. Neratinib inhibits HER-family signaling pathways that promote tumor growth and survival, so increased local drug availability is expected to decrease proliferation and mitotic activity. The microneedle system amplified this effect by optimizing the transdermal deposition of NTB-OOs, while the vesicular carrier allowed the drug to disperse and interact with tumor tissue. Similar results were reported in a study of Ehrlich solid carcinoma treated with nano-gefitinib, showing a significant decrease in mitotic and giant tumor cells and an increase in apoptosis [148]. Thus, the optimized microneedle formulation showed greater inhibition of tumor proliferation than free NTB and the NTB-OOs gel (Figure 11b). The decreased mitotic count and increased necrotic area shown in Figure 11a strongly support the improved antitumor activity of NTB-OOs/PVA-PVP–propolis-MNs, as evidenced histopathological.

## 4. Conclusions

In this study, a novel transdermal platform comprising neratinib-loaded *Ocimum basilicum* oil nanovesicles (NTB-OOs) in PVA/PVP–propolis dissolving microneedles was successfully formulated and evaluated. GC–MS analysis characterized the chemical profile of *Ocimum basilicum* oil, revealing a volatile composition dominated by oxygenated bioactive constituents. Eight major compounds were identified, with Cyclopenta[g]-2-benzopyran, 1,3,4,6,7,8-hexahydro-4,6,6,7,8,8-hexamethyl-, linalool, methyl jasmonate, and linalyl butyrate representing the predominant constituents. The abundance of these metabolites, particularly the oxygenated monoterpene linalool and the jasmonate derivative methyl jasmonate, highlights the potential of *O. basilicum* oil as a multifunctional natural material with antioxidant, anti-inflammatory, and anticancer properties. The optimized NTB-OOs formulation exhibited desirable physicochemical properties, including a particle size of (198.9 ± 0.98 nm), an entrapment efficiency of (88.9 ± 0.45%), a narrow polydispersity index of (0.16 ± 0.01), and a zeta potential of (−23.1 ± 0.35 mV). Morphological, spectroscopic, and thermal analyses confirmed the successful encapsulation of NTB into uniform nanovesicles, reduced drug crystallinity, and no apparent incompatibility among formulation components. Moreover, the optimized nanovesicles maintained physicochemical stability for 6 months, supporting the stabilizing roles of TPGS, cholesterol, and *Ocimum* oil. Incorporation into the microneedle matrix produced mechanically robust, rapidly dissolving arrays with controlled NTB release and significantly improved skin permeation. The improved system increased cumulative NTB permeation to ~3500 µg/cm^2^ and decreased the IC_50_ for MCF-7 cells from 11.1 to 5.1 µg/mL. In Ehrlich solid carcinoma-bearing mice, NTB-OOs/P-MNs led to about 75.6% inhibition of tumor growth, increased mean survival from 19 days to 26.5 days, and produced significant tumor necrosis. The formulation also improved caspase-3-mediated apoptosis and reduced the expression of HER-2, EGFR, VEGF-A, and Bcl-2, while improving inflammatory, oxidative stress, hepatic, renal, and lipid biomarkers. The multifunctional nanovesicle–microneedle system improved NTB stability, controlled release, promoted transdermal delivery, and enhanced antitumor efficiency. This indicates a promising minimally invasive approach for localized breast cancer treatment, but further studies on pharmacokinetics, dermal safety, and long-term efficacy are needed before clinical application. While the formulation improved ex vivo transdermal absorption and in vivo anti-tumor efficacy, precise measurement of localized tumor targeting was not performed. Future studies should assess NTB skin retention and layer-specific deposition, plasma pharmacokinetics, and tissue/tumor biodistribution to evaluate the extent of localized versus systemic drug exposure.

## Figures and Tables

**Table 1:** Custom I-optimal design utilized for optimizing neratinib-loaded *Ocimum* oil nanovesicle formulations (NTB-OOs).

Factors (Independent Variables)	Design Levels		
	Low (−1)	Medium (0)	High (+1)
A: Edge activator amount(mg)	25	50	100
B: Cholesterol amount (mg)	30	60	90
C: Edge activator types	TPGS	Tween 80	sodium cholate
Responses (Dependent variables)	Goal		
Y1: E.E (%)	Maximize		
Y2: PS (nm)	Minimize		
Y3: PDI (nm)	Minimize		
Y4: ZP (mV)	Maximize		

Abbreviations: Entrapment efficiency; PS, Particle size; PDI, polydispersity index; ZP, zeta potential.

**Table 2:** Experimental runs, independent variables, and measured responses of the I-optimal design for optimizing neratinib-loaded *Ocimum* oil nanovesicle formulations (NTB-OOs) (n = 3 ± SD).

Run	Factors			Responses			
	A: Edge Activator Amount (mg)	B: Cholesterol Amount (mg)	C: Edge Activator Type	EE (%)	PS (nm)	PDI	ZP (mV)
1	50	60	TPGS	88.6 ± 0.34	196.4 ± 0.65	0.18 ± 0.01	−21.6 ± 0.56
2	100	30	Tween 80	74.8 ± 0.56	218.3 ± 0.61	0.24 ± 0.04	−11.4 ± 0.84
3	50	60	Sodium cholate	72.5 ± 0.394	203.7 ± 0.95	0.21 ± 0.03	−38.9 ± 0.09
4	25	60	Tween 80	73.1 ± 0.65	258.6 ± 0.87	0.31 ± 0.05	−8.6 ± 0.35
5	50	30	Tween 80	76.4 ± 0.23	226.9 ± 0.45	0.25 ± 0.07	−10.1 ± 0.82
6	50	90	TPGS	90.2 ± 0.94	222.4 ± 0.67	0.24 ± 0.05	−19.8 ± 0.98
7	100	60	Sodium cholate	66.9 ± 0.52	191.6 ± 0.83	0.2 ± 0.01	−44.8 ± 0.45
8	50	30	TPGS	84.1 ± 0.48	188.7 ± 0.84	0.2 ± 0.04	−18.4 ± 0.76
9	50	60	Sodium cholate	73.2 ± 0.24	205.4 ± 0.25	0.22 ± 0.03	−39.5 ± 0.77
10	50	90	TPGS	89.7 ± 0.75	224.1 ± 0.45	0.25 ± 0.04	−20.4 ± 0.56
11	100	90	Sodium cholate	69.4 ± 0.47	218.8 ± 0.86	0.25 ± 0.03	−46.2 ± 0.34
12	50	90	Tween 80	82 ± 0.56	253.5 ± 0.34	0.29 ± 0.02	−13.3 ± 0.55
13	25	30	Sodium cholate	64.5 ± 0.94	239.7 ± 0.56	0.3 ± 0.01	−29.8 ± 0.65
14	50	60	Sodium cholate	72.1 ± 0.95	204.6 ± 0.73	0.22 ± 0.01	−39.1 ± 0.96
15	25	90	Sodium cholate	70.8 ± 0.76	273.2 ± 0.88	0.35 ± 0.05	−32.5 ± 0.46
16	100	60	TPGS	85.7 ± 0.48	178.9 ± 0.45	0.17 ± 0.07	−23.5 ± 0.76
17	50	90	Tween 80	81.4 ± 0.98	251.9 ± 0.76	0.3 ± 0.06	−12.7 ± 0.55
18	100	30	Sodium cholate	63.2 ± 0.91	184.6 ± 0.87	0.19 ± 0.03	−42.1 ± 0.49
19	100	60	Tween 80	76.9 ± 0.34	207.5 ± 0.81	0.22 ± 0.05	−14.8 ± 0.96
20	100	30	TPGS	82.3 ± 0.58	172.8 ± 0.57	0.18 ± 0.03	−22.1 ± 0.88
21	50	60	Sodium cholate	72.8 ± 0.48	204.1 ± 0.76	0.21 ± 0.01	−39.3 ± 0.98
22	25	60	TPGS	82.5 ± 0.91	230.4 ± 0.67	0.25 ± 0.03	−17.5 ± 0.78
OF	68	55	TPGS	88.9 ± 0.45	198.9 ± 0.98	0.16 ± 0.01	−23.1 ± 0.35

Abbreviations: PS, Particle size; PDI, polydispersity index; ZP, zeta potential; and OF, optimized formula.

**Table 3:** Fabrication of PVA/PVP–Propolis Dissolving Microneedles loading freeze-dried neratinib Loaded *Ocimum* Oil Vesicles (NTB-OOs).

Formulations	PVA (% *w*/*w*)	PVP K90 (% *w*/*w*)	Propolis (% *w*/*w*)
Propolis-MNs1	20	25	0.5
Propolis-MNs2	25	20	1
Propolis-MNs3	30	15	1.5

Abbreviation: polyvinyl alcohol (PVA); PVP K90, polyvinyl pyrrolidone K90; MNs, microneedles; NTB, Neratinib; OOs, *Ocimum* Oil Vesicles.

**Table 4:** The primer sequences used for amplification of the mouse HER-2, EGFR, VEGF-A and Bcl-2 genes.

Gene	Primer Sequence (5′ → 3′)	NCBI Reference Sequence	Amplicon Size	Annealing Temperature
*Erbb2 (HER2)*	F: TCGCTCACAACCGAGTGAAA R: ACAGCCAGGGCATACTTGTC	NM_001003817.1	91 bp	60 °C
*EGFR*	F: TGCCAATAATGTCTGCCACCT R: CACTGCCATTGAACGTACCC	NM_007912.4	108 bp	60 °C
*VEGF-A*	F: GCAGACTATTCAGCGGACTCA R: CCGTTGGCACGATTTAAGAGG	NM_001025257.3	146 bp	60 °C
*Bcl-2*	F: AACATCGCCCTGTGGATGAC R: AAACAGAGGTCGCATGCTGG	NM_009741.5	117 bp	60 °C
*GAPDH*	F: AAGGTCGGTGTGAACGGATT R: GAGGTCAATGAAGGGGTCGT	NM_008084.4	108 bp	60 °C

Note: HER-2: Human epidermal growth factor receptor 2; EGFR: Epidermal growth factor receptor; VEGF-A: Vascular endothelial growth factor A; Bcl-2: B-cell lymphoma 2; GAPDH: Glyceraldehyde-3-phosphate dehydrogenase; used as the housekeeping/internal control gene; F: forward; R: reverse.

**Table 5:** Selected constituents identified by GC–MS analysis of *Ocimum basilicum* essential oil.

Peak in Figure	RT (min)	Compound	Molecular Formula	Area	Relative Peak Area (%)
1	12.325	2-Octanol, 2-methyl-	C_9_H_20_O	1,048,728.47	0.22
2	14.897	Linalool	C_10_H_18_O	9,265,844.69	1.97
3	17.992	Linalyl butyrate	C_14_H_24_O_2_	1,941,347.61	0.41
4	21.607	2(3H)-Furanone, dihydro-5-pentyl-	C_9_H_16_O_2_	10,013,135.40	2.13
5	26.159	Cyclopentaneacetic acid, 3-oxo-2-pentyl-, methyl ester	C_13_H_22_O_3_	64,596,883.10	13.74
6	26.621	Methyl jasmonate	C_13_H_20_O_3_	11,822,956.00	2.51
7	28.869	Cyclopenta[g]-2-benzopyran, 1,3,4,6,7,8-hexahydro-4,6,6,7,8,8-hexamethyl-	C_18_H_26_O	91,732,148.10	19.51

Note: Rt = Retention time.

**Table 6:** Analysis of variance (ANOVA) table for optimizing neratinib-loaded *Ocimum oil nanovesicle* formulations (NTB-OOs) with their measured responses (n = 3 ± SD).

Responses	Y1: EE%	Y2: PS	Y3: PDI	Y4: ZP
Minimum	63.2	178.9	0.17	−46.2
Maximum	90.2	273.2	0.35	−8.6
Model	Quadratic	Quadratic	Quadratic	Quadratic
*F*-value	485.00	78.34	95.32	963.20
*p*-value	<0.0001	<0.0001	<0.0001	<0.0001
R^2^	0.998	0.989	0.9906	0.9991
Adjusted R^2^	0.996	0.976	0.9802	0.9980
Predicted R^2^	0.985	0.769	0.9247	0.9913

**Table 7:** Stability study of the optimized neratinib-loaded *Ocimum* oil nanovesicle formulation (NTB-OOs).

Storage Time	Refrigerated Temperature (4 ± 1 °C)				Ambient Temperature			
	EE (%)	PS (nm)	PDI (nm)	ZP (mV)	EE (%)	PS (nm)	PDI (nm)	ZP (mV)
After 24 h	88.9 ± 0.45	198.9 ± 0.98	0.16 ± 0.01	−23.1 ± 0.35	87 ± 0.98	198 ± 0.98	0.19 ± 0.08	−22 ± 0.98
3 months	85 ± 0.99	202 ± 0.47	0.19 ± 0.08	−20.3 ± 0.12	83 ± 0.65	209 ± 0.05	0.21 ± 0.07	−19 ± 0.82
6 months	81 ± 0.65	210 ± 0.87	0.21 ± 0.02	−19.1 ± 0.41	80 ± 0.98	214 ± 0.02	0.23 ± 0.04	−19 ± 0.18

Data are displayed as mean ± SD from three independent tests (n = 3).

**Table 8:** Kinetic analysis of the release behavior of neratinib from the optimum neratinib-loaded *Ocimum* oil nanovesicles (NTB-OOs), its corresponding PVA/PVP–Propolis Dissolving Microneedles loading freeze-dried neratinib-loaded *Ocimum* oil vesicles (NTB-OOs), and neratinib suspension.

Formulation	Zero Order (R^2^)	First Order (R^2^)	Higuchi (R^2^)	Korsmeyer–Peppas (R^2^)	n (Peppas)	Best Fit Model
NTB Free OOs	0.852	0.978	0.945	0.961	0.682	First Order
NTB-OOs	0.891	0.943	0.987	0.989	0.524	Korsmeyer–Peppas
NTB-OOs loaded Propolis-MNs	0.986	0.921	0.968	0.973	0.892	Zero Order

Note: NTB Free OOs: neratinib suspension; NTB-OOs: optimum neratinib-loaded *Ocimum* oil nanovesicles; NTB-OOs loaded Propolis-MNs: PVA/PVP–Propolis Dissolving Microneedles loaded with freeze-dried NTB-OOs.

**Table 9:** Effect of different treatments on liver and kidney function biomarkers.

Group	AST (U/L)	ALT (U/L)	ALP (U/L)	Total Bilirubin (mg/dL)	Urea (mg/dL)	Creatinine (mg/dL)	Uric Acid (mg/dL)
ESC model	136.7 ± 7.9	72.8 ± 5.4	164.3 ± 8.6	0.89 ± 0.07	61.8 ± 4.7	0.89 ± 0.07	4.8 ± 0.4
NTB gel	118.5 ± 6.8	61.4 ± 4.2	143.6 ± 7.2	0.74 ± 0.06	53.4 ± 3.9	0.76 ± 0.06	3.9 ± 0.3
NTB-OOs gel	96.3 ± 5.7	48.7 ± 3.6	119.4 ± 6.5	0.58 ± 0.05	43.7 ± 3.2	0.61 ± 0.05	3.0 ± 0.3
NTB-OOs/P-MNs	86.9 ± 4.9	40.8 ± 3.1	104.7 ± 5.8	0.48 ± 0.04	36.8 ± 2.7	0.52 ± 0.04	2.4 ± 0.2

Abbreviations: ESC (Ehrlich carcinoma model, untreated), GII (Neratinib gel with 2% *w*/*w* HPMC), GIII (NTB-OOs in gel with 2% *w*/*w* HPMC), and GIV (NTB-OOs/PVA-PVP Propolis-MNs).

**Table 10:** Effect of different treatments on lipid profile.

Group	TG (mg/dL)	TC (mg/dL)	HDL-C (mg/dL)	LDL-C (mg/dL)	VLDL-C (mg/dL)
ESC model	152.7 ± 8.6	168.5 ± 9.4	28.6 ± 2.1	109.4 ± 6.7	30.5 ± 1.7
NTB gel	131.4 ± 7.5	145.2 ± 8.1	33.8 ± 2.5	85.1 ± 5.3	26.3 ± 1.5
NTB-OOs gel	108.6 ± 6.3	121.8 ± 7.2	39.7 ± 2.9	60.4 ± 4.6	21.7 ± 1.3
NTB-OOs/P-MNs	91.7 ± 5.8	103.6 ± 6.5	44.2 ± 3.1	41.1 ± 3.8	18.3 ± 1.2

Abbreviations: ESC (Ehrlich carcinoma model, untreated), GII (Neratinib gel with 2% *w*/*w* HPMC), GIII (NTB-OOs in gel with 2% *w*/*w* HPMC), and GIV (NTB-OOs/PVA-PVP Propolis-MNs).

## Data Availability

The raw data supporting the conclusions of this article will be made available by the authors on request.

## Abbreviations

Abbreviation	Full term
AKT	Protein kinase B
ALP	Alkaline phosphatase
ALT	Alanine aminotransferase
ANOVA	Analysis of variance
ARRIVE	Animal Research: Reporting of In Vivo Experiments
AST	Aspartate aminotransferase
ATCC	American Type Culture Collection
BC	Breast cancer
Bcl-2	B-cell lymphoma 2
BCS	Biopharmaceutics Classification System
CAT	Catalase
CV	Cell viability
DOX	Doxorubicin
DSC	Differential scanning calorimetry
EAC	Ehrlich ascites carcinoma
EDTA	Ethylenediaminetetraacetic acid
EE	Entrapment efficiency
EGFR	Epidermal growth factor receptor
ELISA	Enzyme-linked immunosorbent assay
ESC	Ehrlich solid carcinoma
FTIR	Fourier-transform infrared spectroscopy
GAPDH	Glyceraldehyde-3-phosphate dehydrogenase
GC–MS	Gas chromatography–mass spectrometry
H&E	Hematoxylin and eosin
HDL	High-density lipoprotein
HDL-C	High-density lipoprotein cholesterol
HER	Human epidermal growth factor receptor
HER-2	Human epidermal growth factor receptor 2
HPLC–UV	High-performance liquid chromatography with ultraviolet detection
HPMC	Hydroxypropyl methylcellulose
IC_50_	Half-maximal inhibitory concentration
ICH	International Council for Harmonisation
IL-6	Interleukin-6
ILS	Increase in life span
LFTs	Liver function tests
LOD	Loss on drying
MAPK	Mitogen-activated protein kinase
MCF-7	Michigan Cancer Foundation-7 human breast adenocarcinoma cell line
MDA	Malondialdehyde
MNs	Microneedles
mRNA	Messenger ribonucleic acid
MST	Mean survival time
NTB	Neratinib
NTB-OOs	Neratinib-loaded *Ocimum* oil nanovesicles
NTB-OOs/P-MNs	Neratinib-loaded *Ocimum* oil nanovesicles incorporated into propolis dissolving microneedles
OF	Optimized formula
OO	*Ocimum* oil
OOs	*Ocimum* oil nanovesicles
PDI	Polydispersity index
PDMS	Polydimethylsiloxane
PEG	Polyethylene glycol
PS	Particle size
PVA	Polyvinyl alcohol
PVP	Polyvinylpyrrolidone
qRT-PCR	Quantitative real-time polymerase chain reaction
SEM	Scanning electron microscopy
TEM	Transmission electron microscopy
TG	Triglycerides
TNF-α	Tumor necrosis factor alpha
TPGS	D-α-Tocopheryl polyethylene glycol 1000 succinate
VEGF-A	Vascular endothelial growth factor A
ZP	Zeta potential
